# A multidimensional framework to quantify the effects of urbanization on avian breeding fitness

**DOI:** 10.1002/ece3.10259

**Published:** 2023-07-03

**Authors:** Sihao Chen, Yu Liu, Samantha C. Patrick, Eben Goodale, Rebecca J. Safran, Emilio Pagani‐Núñez

**Affiliations:** ^1^ Department of Health and Environmental Sciences Xi'an Jiaotong‐Liverpool University Suzhou China; ^2^ Department of Earth, Ocean and Ecological Sciences, School of Environmental Sciences University of Liverpool Liverpool UK; ^3^ Key Laboratory for Biodiversity Science and Ecological Engineering, Ministry of Education, College of Life Sciences Beijing Normal University Beijing China; ^4^ Department of Ecology and Evolutionary Biology University of Colorado Boulder Colorado USA; ^5^ School of Applied Sciences Edinburgh Napier University Edinburgh UK; ^6^ Centre for Conservation and Restoration Science Edinburgh Napier University Edinburgh UK

**Keywords:** artificial light at night, breeding fitness, environmental filter, food resources, land‐use change, life‐history traits, noise pollution, trophic interaction, urban heat island, urbanization

## Abstract

Urbanization has dramatically altered Earth's landscapes and changed a multitude of environmental factors. This has resulted in intense land‐use change, and adverse consequences such as the urban heat island effect (UHI), noise pollution, and artificial light at night (ALAN). However, there is a lack of research on the combined effects of these environmental factors on life‐history traits and fitness, and on how these interactions shape food resources and drive patterns of species persistence. Here, we systematically reviewed the literature and created a comprehensive framework of the mechanistic pathways by which urbanization affects fitness and thus favors certain species. We found that urbanization‐induced changes in urban vegetation, habitat quality, spring temperature, resource availability, acoustic environment, nighttime light, and species behaviors (e.g., laying, foraging, and communicating) influence breeding choices, optimal time windows that reduce phenological mismatch, and breeding success. Insectivorous and omnivorous species that are especially sensitive to temperature often experience advanced laying behaviors and smaller clutch sizes in urban areas. By contrast, some granivorous and omnivorous species experience little difference in clutch size and number of fledglings because urban areas make it easier to access anthropogenic food resources and to avoid predation. Furthermore, the interactive effect of land‐use change and UHI on species could be synergistic in locations where habitat loss and fragmentation are greatest and when extreme‐hot weather events take place in urban areas. However, in some instances, UHI may mitigate the impact of land‐use changes at local scales and provide suitable breeding conditions by shifting the environment to be more favorable for species' thermal limits and by extending the time window in which food resources are available in urban areas. As a result, we determined five broad directions for further research to highlight that urbanization provides a great opportunity to study environmental filtering processes and population dynamics.

## Graphical abstract

Across the last decades, there has been an increasing interest in determining how urbanization shapes wildlife ecology and behavior. Here, we conducted a systematic review on studies assessing the effect of urbanization on avian breeding fitness. We produced a multidimensional framework integrating potential environmental factors to assess the synergistic effects of these factors on food availability and fitness of urban birds.

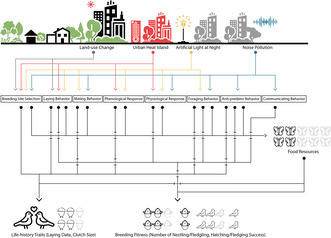

## 1 INTRODUCTION

### 1.1 Urbanization and biodiversity

The impacts of urbanization on biodiversity and ecosystems will increase exponentially across the twenty‐first century as regions such as Africa, Asia, and Latin America increase their urban populations from 3.23 to 5.56 billion people (2018–2050), whereas developed countries in Europe and Northern America will experience a slight change of about 0.81% increase annually (United Nations, 2019). This explosive population increase, particularly in Africa, Asia, and Latin America, requires land to be converted to urban areas. The surface of natural habitats lost to urbanization will reach 10^5^ km^2^ by 2050 (Li et al., 2022). As a consequence, it will cause an estimated 13.6% reduction in species richness and a 10.7% reduction in the abundance of vertebrates, invertebrates and plants by 2050 globally (Newbold et al., 2015). This will contribute further to a 34% decrease of common vertebrates' species richness and a 52% decrease of species abundance by 2100 under the current trajectory, which adds to the 70% decrease of vertebrate abundance over the last 50 years across continents (Li et al., 2022; WWF, 2022). This loss indicates that urbanization has a strong impact on biodiversity. Therefore, systematic analysis of aspects of the urbanization process that are more harmful for wildlife is central to avert further biodiversity loss.

### 1.2 Urban ecosystem and filtering

**FIGURE 1:**
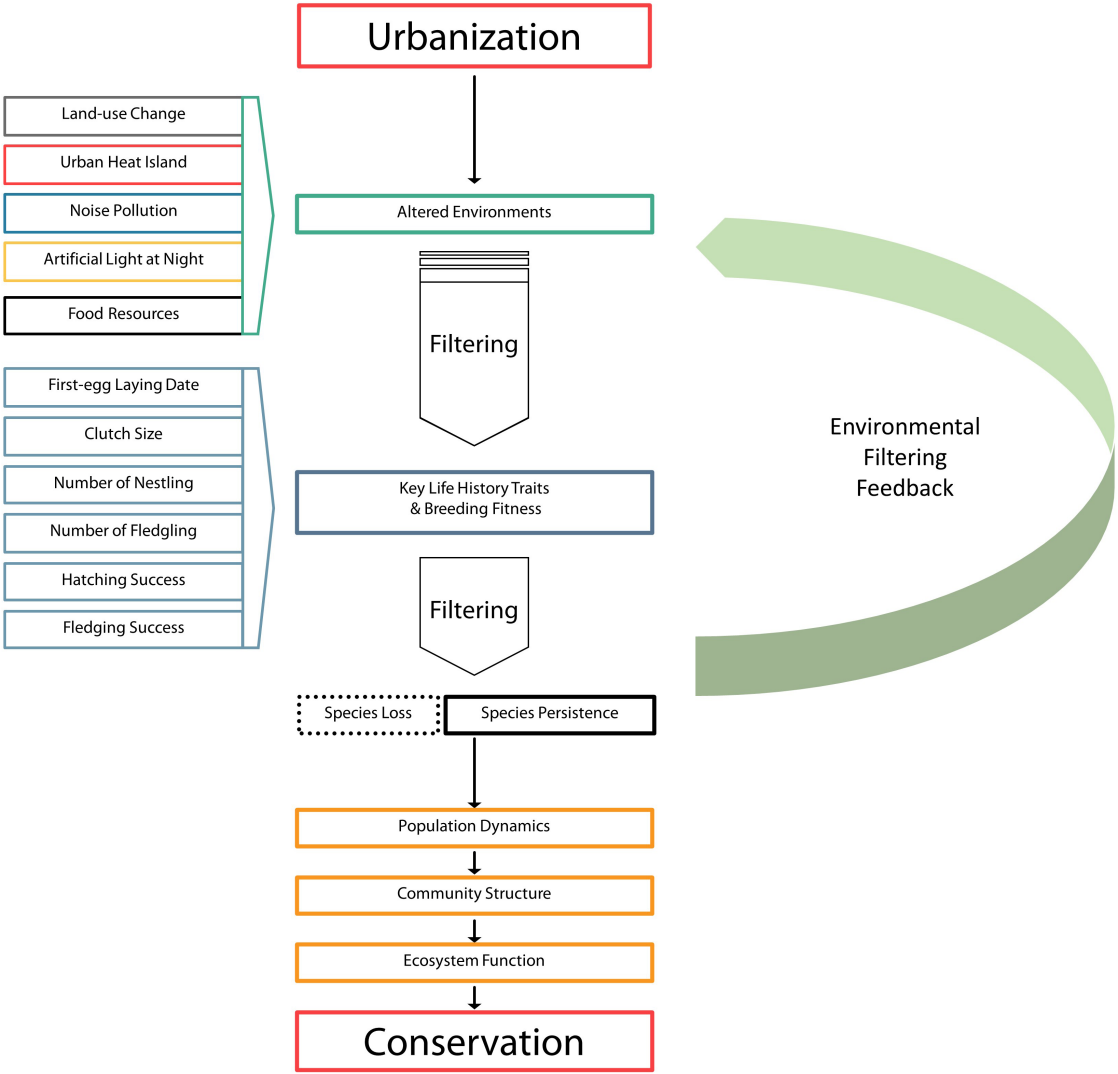
Conceptual framework illustrating how urbanization can drive changes in species' persistence or loss, and in turn influences conservation efforts through the individual‐to‐community dynamics. The general flow of the framework is adapted from Alberti (2015).

Species loss due to urbanization is largely attributed to the fact that urban habitats are novel ecosystems and many species are not able to cope with these rapid changes in their environmental (Aronson et al., 2014; Donihue & Lambert, 2015; Futuyma, 2005; Thompson et al., 2022). In the early 1990s, Keddy (1992) defined habitats and their associated environmental features as filters that determine assembly rules driven by directional selection, and this concept has also been applied to urban environments. Here, we refer to filtering as the favorable outcome of higher reproductive success and breeding fitness in urban areas as (Figure 1). As such, species able to persist in urban areas possess particular genotypes, functional (e.g., behavioral and physiological), and life‐history (e.g., phenological and reproductive) traits enabling them to outcompete other species (Martin & Bonier, 2018; Thompson et al., 2022; Violle et al., 2007). For example, species with high functional plasticity are able to modify foraging, offspring provisioning, and communication facilitating colonization and persistence in novel urban environments (Kight & Swaddle, 2011; Lowry et al., 2013; Russ et al., 2017; Wang et al., 2021). Species with high physiological tolerance may build up resistance to circadian and metabolic disruption and abnormal oxidative stress in urban environments (Dominoni et al., 2013; Gaston et al., 2013; Navara & Nelson, 2007). Finally, species with high reproductive plasticity may cope with environmental changes by advancing laying dates and laying smaller clutch sizes. However, our understanding of how urbanization and associated environmental changes act as a species filter requires synthesis. Previous research has placed great emphasis in ascertaining which traits are linked to species persistence in urban environments (Gil & Gahr, 2002; Lowry et al., 2013; Palkovacs et al., 2012), but a comprehensive framework of the mechanistic pathways by which urbanization affects fitness and thus favors certain species is lacking (Holt & Comizzoli, 2022; Thompson et al., 2022).

#### 1.2.1 Potential urban filters

This gap in knowledge for how animals fare in urbanized habitats is understandable because potential filters such as the urban heat island (UHI) effect (Zhao et al., 2014), noise pollution (Francis & Barber, 2013; Kleist et al., 2018), and artificial light at night (ALAN) (Gaston et al., 2013) co‐occur with land‐use change (changes in land‐use cover for human uses and increased impervious surfaces; Aronson et al., 2014; Grimm et al., 2008; Sih et al., 2011), making it difficult to tease apart their effects on fitness (Holt & Comizzoli, 2022). Here, we refer to the UHI effect as the differences in surface and air temperature between urban centers and peri‐urban areas (Oke, 1995). The most important contributor to this effect is the predominance of impervious surfaces, which absorb solar radiation and anthropogenic heat, accounting for 70% of the temperature increase in urban centers (Imhoff et al., 2010). For example, daytime surface temperature can increase up to 7°C in cities (e.g., Medellín and Tokyo) compared with peri‐urban areas, while this difference is narrowed down to ~2°C at night (Peng et al., 2012). Noise pollution is usually generated by human activities including land development (81–113 dB ambient noise level) and transportation networks (80–120 dB) depending on noise frequency (Ouis, 2001), and has increased its magnitude and extent dramatically across the last decades (Shannon et al., 2016). In the United States, for example, roadway and airway traffic volume have tripled since the early 1980s (Barber et al., 2010). Similarly, ALAN pollution is widespread. The number of people living under a night sky affected by human light pollution has increased from 40% of the world's population in 2001 to 83% in 2016 (Cinzano et al., 2001; Falchi et al., 2016). Additionally, indirect light exposure (i.e., artificial skyglow) can affect vast areas and make the nighttime light level in urban areas increase up to four orders of magnitude compared to natural environments (Kyba et al., 2015). While these impacts are pervasive, it is currently difficult to determine which combinations of them are the most harmful for wildlife.

#### 1.2.2 Effect of urban filters on species

To determine which environmental factors act as a filter for different species across urban gradients, we examined how land‐use change, the UHI effect, noise pollution and ALAN, separately and combined, impact food resources, life‐history traits, and breeding fitness. We focused on avian taxa because there is a wealth of information documenting the effects of urbanization on multiple life‐history traits and fitness dimensions for birds (Chamberlain et al., 2009; Swaddle et al., 2015; Visser & Gienapp, 2019), and because they are particularly sensitive to urbanization and global change (Bowler et al., 2019; Rosenberg et al., 2019). Birds are thus a perfect system to study the specific mechanisms through which urbanization filters species. These relationships are extremely complex; it is therefore necessary to assemble an integrative framework incorporating potential environmental filters, resource availability, life‐history traits, and fitness characteristics across its multiple dimensions. It is widely accepted that these avian life‐history traits are tightly linked to urban‐associated environmental factors. For example, avian species generally have been reported to consistently lay their eggs earlier, and produce smaller clutches, and reduced numbers of nestlings and fledglings in urban landscapes (Capilla‐Lasheras et al., 2022; Chamberlain et al., 2009; Sepp et al., 2018). Furthermore, species have been shown to respond to the UHI effect, noise pollution, and ALAN. As an example of the UHI effect, with an average of 2.3 days of spring advancement per decade, 78 out of 168 bird species have advanced their laying date while the rest show delayed (14) or no change (76) (Parmesan & Yohe, 2003) in laying dates. Advanced laying dates can have a negative effect on fitness if they lead to mismatches between the time of breeding and availability of resources, but they could also have a positive effect if they allow time for more clutches within a single breeding season (Futuyma, 2005; Visser & Gienapp, 2019). As an example of noise pollution, anthropogenic noise that overlaps with the acoustic niche (1–5 kHz) of Eastern Bluebirds *Sialia sialis* results in a reduction of up to three fledglings (Kight et al., 2012), while low‐frequency noise (68 dB measured at the entrance to the nest box) reduces fledging success as much as 20% in House Sparrows *Passer domesticus* compared to quiet environments (50 dB; Schroeder et al., 2012). Finally, as an example of ALAN, under the presence of streetlights, females of Blue Tits *Cyanistes caeruleus*, Great Tits *Parus major*, Blackbirds *Turdus merula*, and European Robins *Erithacus rubecula* start egg laying 1.5 days earlier on average than without artificial light sources (Kempenaers et al., 2010). Moreover, 13 of 27 species experienced strong negative responses to ALAN, while 16 species able to exploit opportunistically niches created by artificial light produced up to 16% larger clutch sizes (Senzaki et al., 2020).

Here, we considered multiple life‐history traits and fitness components, including: first egg‐laying date (clutch initiation), clutch size (number of eggs laid per attempt), number of nestlings/fledglings (hatched and fledged individuals), and hatching/fledging success (ratio of number of nestlings to clutch size and fledglings to nestlings). These indicators of reproductive success and breeding fitness are widely used in ecology, and have been documented across many different taxa in similar studies (Chamberlain et al., 2009; Futuyma, 2005). We use “reproductive success” to refer to number of fledglings and “breeding fitness” to refer to all reproductive parameters (i.e., hatching/fledging success and number of hatchlings/fledglings).

#### 1.2.3 Effect of urban filters on food resources

Environmental factors can not only have a direct effect on breeding fitness but also produce indirect impacts by altering food resources. Food resources play a vital role in shaping the interactions between trophic levels and has long been considered a key factor shaping breeding fitness of animals (White, 2008). Interestingly, urbanization dramatically alters trophic webs in complex ways, which may result in positive and negative effects for different species (Ockendon et al., 2014; Renner & Zohner, 2018). For example, high urbanization intensity leads to an approximately 20% plant species richness and 15% abundance loss compared with cities of low urbanization intensity (Newbold et al., 2015). Thus, species feeding on plant food resources may be negatively affected by land‐use change. In addition, urban areas are often associated with more non‐native plant species, and these species have been linked with lower arthropod abundance and food quality (Aronson et al., 2015; Narango et al., 2018). However, species that can exploit urban plants may display positive responses due to the UHI effect. For instance, land surface temperature has led to a net increase of enhanced vegetation growth by 15 days in eastern North American cities compared to nonurban areas (Zhang et al., 2004).

Insects as food resources may also respond differently to urbanization. On one hand, land‐use change can reduce invertebrate species richness by 43% and abundance by 60% (Millard et al., 2021) and the UHI effect can exceed the thermal limits of ectotherms (Huey et al., 2012). On the other hand, land‐use change and the UHI effect may indirectly ameliorate food scarcity by favoring generalist invertebrates through the massive implementation of monotonic city greening and increased temperatures (Meineke et al., 2013). Noise pollution can change the behavior and physiology of invertebrates, affecting their mating and reproductive success (Classen‐Rodríguez et al., 2021). For example, background compressor noise (55 dB measured at 50 m, frequencies ranging from 20 to 5000 Hz) can reduce the abundance of Acrididae, Cercopidae, and Rhaphidophoridae families, with effects ranging from 24% to 95% decreases in their population sizes (Bunkley et al., 2017), and these insect families are important food resources for certain bird species (Carlisle et al., 2012; Gámez‐Virués et al., 2007; Kleintjes & Dahlsten, 1994). Additionally, ALAN can reduce local insect abundance by 33%–47% under light‐emitting diodes (LED) and high‐pressure sodium lumps (HPS) (Boyes et al., 2021). However, noise pollution may contribute towards increasing the concentrations of insects even in relatively quiet urban areas (Bunkley et al., 2017; Mazzoni et al., 2009), where species may benefit from the foraging opportunities provided by artificial light (Russ et al., 2015).

#### 1.2.4 Combined effects of urban filters

As environmental changes overlap in space and time, there may be noncumulative (additive effects in which factors affect species separately but with an effect equal to the sum of individual effects) and cumulative effects (factors affecting species either antagonistically, with an effect offsetting the other, or synergistically, with an effect exacerbating the other additive effects) on life‐history traits and breeding fitness (Galic et al., 2018). For example, 59 out of 108 bird species have been reported to be impacted by synergistic effects of land‐use change and climate change, experiencing long‐term population declines, with insectivores experiencing stronger declines than granivores (Betts et al., 2019). Conversely, there are studies illustrating a weak synergistic effect of land‐use change and climate change on the number of fledglings (<0.43 fledgling difference), suggesting that these synergistic effects may not be widespread (Saunders et al., 2021). A study conducted in North America found that the abundance of 40% and 28% of 140 avian species decreased due to noise pollution and ALAN, respectively, while the number of affected species increased up to 70% as a result of synergistic effects by both factors (Wilson et al., 2021). However, an experiment on Western Bluebirds *Sialia mexicana* reported surprising patterns. When compared with nests exposed to approximately 65 dB noise and 3.3 lux light illumination inside the nest box, nests under the “only noise” treatment produced one additional fledgling compared to control groups (i.e., no noise or light) and performed much better compared to only light‐lit groups (Ferraro et al., 2020). These contradictory results between model simulations and empirical research suggest that a comprehensive multidimensional framework is required to fully understand these complex interactions.

### 1.3 Aim of the research

**FIGURE 2:**
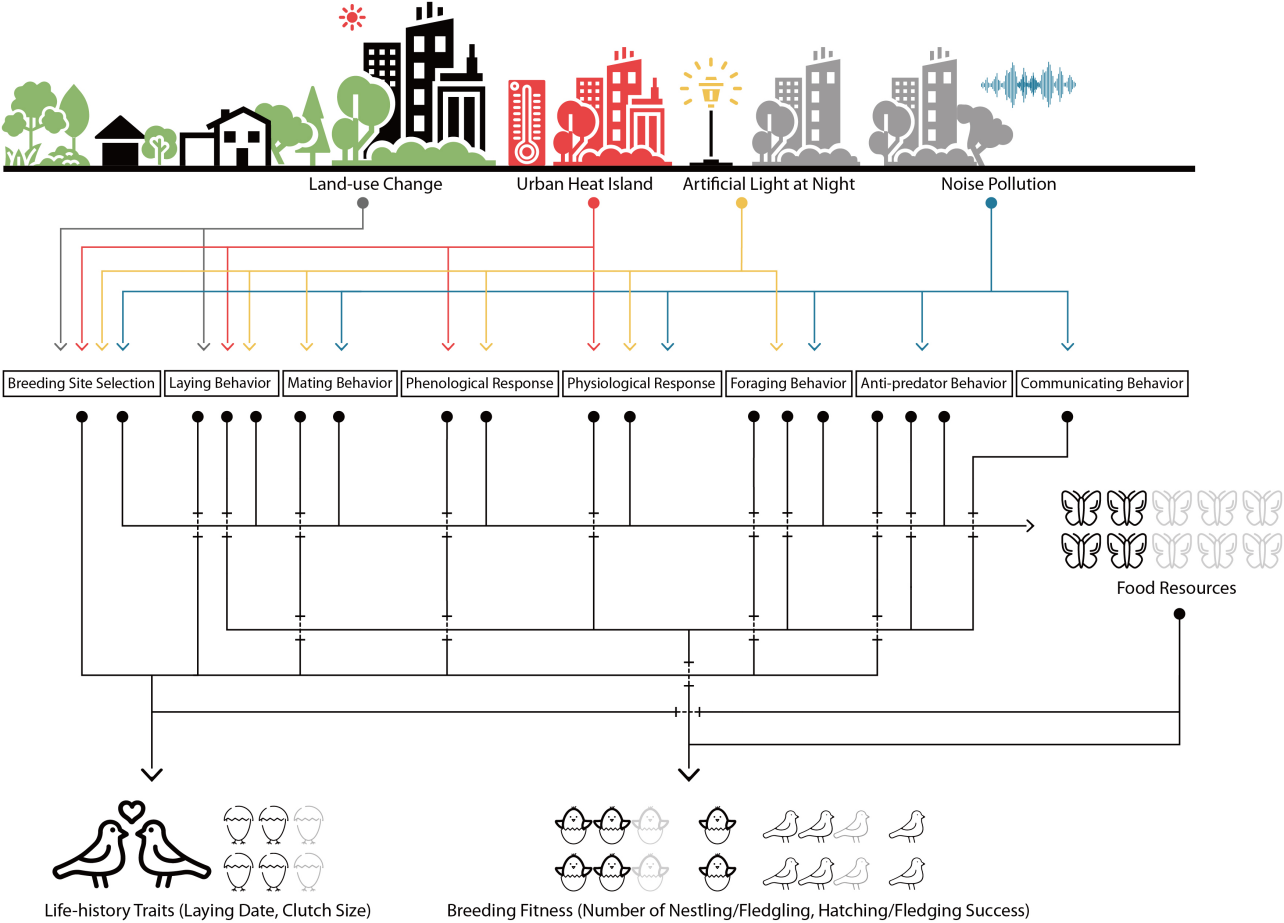
Conceptual framework illustrating how urbanization and its associated factors can drive interactive changes in food resources, life‐history traits, and breeding fitness. This framework consists of four horizontal sections. The first section on top depicts four environmental factors. The second section with box around depicts potential mechanisms through which environmental factors can affect either food resources or species (i.e., fourth section). Food resources, as a mediator, is the third section, through which environmental factors and species are linked. Specifically, urbanization‐related land‐use changes such as conversion of natural land to built‐up areas can not only be linked to decreased habitat quality and productivity (Breeding Site Selection), and reduced insect abundance, but also elevated ambient temperature urban heat island (UHI). Increased temperatures may increase insect populations, but also result in phenological mismatches between the bird–insect–plant food chain and disrupt the timing of incubation (laying and mating behavior, and phenological response). Noise pollution may decrease insect abundance via interfering with courtship and reproduction processes and drive abnormal physiological responses of birds and their offspring (laying and mating behavior, physiological response, and foraging and communicating behavior). Artificial light at night may disrupt circadian rhythms by reducing sleeping time and negatively affecting breeding fitness and long‐term individual survival (physiological response), whereas extended light at nighttime would also enable breeding individuals to forage longer and attract common insect species, therefore, providing more food to their offspring (foraging behavior). Light gray icons represent potential food loss or breeding cost due to environmental factors, in comparison to the absence of these factors. The framework is conceptualized by authors, and icons adapted from NounProject.com (CC BY 3.0, Appendix S4: Table S1).

The best studied interactions are between land‐use and climate change (used here as a proxy of the UHI effect), and noise pollution and ALAN (Halfwerk & Jerem, 2021; Mantyka‐Pringle et al., 2012; Wilson et al., 2021; Zhao et al., 2022). However, these studies paid more attention to either interactive mechanisms (Dominoni, Halfwerk, et al., 2020; Swaddle et al., 2015), species distributions (Sohl, 2014), or community composition (Peterson et al., 2015) than to the effect of urban environmental factors on food resources and on life‐history traits and breeding fitness (Holt & Comizzoli, 2022). More specifically, there are significant research gaps regarding (1) the cumulative and noncumulative effects between land‐use change, UHI effect, noise pollution, and ALAN, and (2) their combined effects on food resources, life‐history traits, and breeding fitness. To fill in these gaps, we conducted a systematic review and synthesized knowledge on single and combined effects of these factors, paying particular attention to how they may affect food resources. In doing so, we constructed a novel multidimensional framework (Figure 2) to assess these complex effects and to answer the following questions: (1) how and why do avian species respond to land‐use change, UHI effect, noise pollution, and ALAN; (2) how and why do potential food resources for birds respond to land‐use change, UHI effect, noise pollution, and ALAN; and (3) what are the synergistic or antagonistic effects of land‐use change, UHI effect, noise pollution, and ALAN on both available food resources and breeding fitness. We then discuss these findings and outline outstanding research questions and knowledge gaps for further investigation.

## 2 METHODS

We used a systematic review approach instead of a meta‐analysis because we found that there was little data available for some factors like UHI, noise pollution, and ALAN, and their interactions (Gurevitch et al., 2018). For land‐use change, 30 studies investigated the relationships between land‐use change and life‐history traits and breeding fitness, but a large proportion of these 30 studies includes studies that had already been synthesized in Chamberlain et al. (2009).

### 2.1 PRISMA protocol

We followed the PRISMA protocol to identify relevant articles (Shamseer et al., 2015). We selected online search engines Web of Science and Scopus to perform a literature search by combining four topic sections (TS) with different keyword strings: (1) TS = (“urban*”) AND (2) TS = (“bird$” OR “avian”) AND (3) TS = (“surviv*” OR “breed*” OR “clutch size$” OR “laying date$” OR “hatching/fledging success” OR “reproduct*”) AND (4) TS = (“noise” OR “sound$” OR “man‐made noise” OR “anthropogenic noise” OR “man‐made sound$” OR “noise pollution” OR “light at night$” OR “anthropogenic light$” OR “light pollution” OR “urban heat island effect$” OR “temperature” OR “food*” OR “prey”).

Publications yielded from Web of Science and Scopus were initially compared, and duplicates were deleted. Two researchers (SC and YL) screened all the titles and abstracts left in the selection pool, and coded whether the publications met the criteria independently (Table 1; Appendix S1: Figure S1). Specifically, based on information contained in the title and abstract, we first determined whether this article studied avian species and then whether the article contained or may contain one or more of environmental factors and life‐history traits/breeding fitness. For factors, we meant at least one pair of comparisons must be presented (i.e., urban vs. non‐urban, high vs. low temperature/noise/light, and more vs. less food) in the study. If these three criteria were met, these articles were marked for further full‐text reviewing. For articles that were coded differently by the two researchers or were in doubt, a full‐text review was performed to determine its inclusion. During the full‐text reviewing process, we excluded articles that (1) did not include at least one of the four studied factors; (2) did not measure reproductive success directly and used age ratios instead; (3) used the same breeding datasets for multiple publications; we only counted one of these articles (generally the one encountered first); and (4) were not related to our criteria even though the titles and abstracts appeared relevant. During this process, review articles were also identified to complement the online research results. Relevant articles cited in these review articles were also examined using the same selection criteria.

**TABLE 1:** Criteria for article refining and selection based on the requirements of PRISMA (Shamseer et al., 2015).

Population	Any avian species
Exposure	Land‐use change, urban heat‐island effect, noise pollution, artificial light at night, and food resources
Comparator	Breeding performance under the influence of different abiotic stressors/factors
Outcome	Breeding metrics including laying dates, clutch sizes, number of nestlings and fledglings, hatching and fledging success rates, and other breeding parameters
Study design	Before–after (BA) and Time‐series (TS)
Timing	2000–2020 inclusive
Document types	Articles and reviews
Language	English only

Afterward, data extraction was performed by a single researcher (SC) with reference to a pre‐determined data extraction template, which was adapted from review articles from our research field. To validate the spreadsheet, the data extraction template was reviewed by all the researchers involved in this study. Specifically, for each article, we collected data related to generic information (i.e., journal information, year of publication, duration of the study, nature of the research, and geographical context), species information (i.e., common and scientific names, number of species studied, habitat information based on ICUN, diet preferences, migratory status, nest shapes, and sample size) (Pigot et al., 2020; Wilman et al., 2014), and effects of studied factors (i.e., urban vs. non‐urban, high vs. low temperature/noise/light, and more vs less food) on life‐history traits (i.e., laying date, clutch size, and number of nestlings/fledglings) and breeding fitness (i.e., hatching/fledging success). We also documented under which environmental conditions species experience earlier laying dates, larger clutch and brood sizes, and higher hatching/fledging success. In total, 80 out of 1129 articles met our review criteria and were scored to obtain information.

### 2.2 Urbanization and monitoring technology

To make studies comparable, we adapted and modified several classification systems. Although it is difficult to quantify studies using a uniform scale, we used very broad categories of urbanization and monitoring technology to illustrate how environmental factors impact fitness. We first adopted a three‐level urbanization scale (Marzluff et al., 2001; Vincze et al., 2017) and 50 papers were classified into urban, peri‐urban, and natural/rural areas (Table 2; Appendix S2: Table S1). Forty‐four articles used the urban and natural/rural category, while nine articles focused on peri‐urban areas. Second, we summarized and grouped articles according to their monitoring technology (Appendix S3: Tables S1–S4). Specifically, nine of 11 articles studying temperature obtained such data from meteorological stations or governmental institutions, and one from dataloggers. Five of 13 articles measuring noise used playback methods to experimentally simulate noise pollution. Seven of these 13 articles used dataloggers to record noise intensity levels. One article used distance to roads as a proxy of noise pollution. In terms of ALAN, five articles employed four methods including light treatments (LED lights; *N* = 1), light meters (*N* = 1), data from governmental institutions (*N* = 2), and online data sources (*N* = 1). Lastly, 25 articles used four approaches to characterize food abundance including food supplementation (*N* = 11), frassfall collection (*N* = 7), pellet collection (*N* = 3), and other unconventional methods (*N* = 4).

**TABLE 2:** A three‐level urbanization scale adapted from Marzluff et al. (2001) and Vincze et al. (2017).

Term	Definition
Natural/Rural	With low or no proportion of built surfaces (<20% within study area), for example, deciduous/coniferous forests, riparian forests, mixed woodland, national/wilderness parks, grasslands, agricultural land, and farmland
Peri‐urban (suburban)	With medium proportion of built surfaces (20%–50% within study area), for example, outskirts, a built‐up area on the periphery of the city/town with open pastures, recreational facilities, and scattered buildings
Urban	With high proportion of built surfaces (>50% within study area), for example, urban/town centers, residences, offices, commercial and industrial land, community, city parks/parklands surrounded by built surfaces, cemeteries surrounded by built surfaces, public golf courses surrounded by built surfaces, and university campuses surrounded by built surfaces

### 2.3 Article categorization

Articles were also classified based on themes, including natural‐to‐urban environments (used here as a proxy of land‐use change), UHI, noise pollution and ALAN, and food resources. Due to a limited number of studies conducted on the relationships between UHI and life‐history traits, we sometimes used articles on climate change to illustrate the relationships between temperature and life‐history traits. All articles fall into at least one theme while some of them belong to two or more themes according to the selection criteria (Table 1). Specifically, 61 of 80 articles analyzed a single environmental factor, while 19 articles analyzed at least two (Table 3). Sixty five percentage of articles investigated either exclusively or partially the impact of land‐use change on breeding performance. Articles involving the study of noise pollution and food resources constituted 47.5%. Only four articles (5%) included ALAN as a study variable. The categorized articles were utilized to identify separate and combined effects of these environmental factors on first egg‐laying date, clutch size, number of nestlings and fledglings, and hatching and fledging success (Table 3).

**TABLE 3:** Number of studies in relation to environmental factors and life‐history traits under each category.

Environmental factors	Laying date^b^	Clutch size^b^	Number of nestlings^b^	Number of fledglings^b^	Hatching success^b^	Fledging success^b^
Land‐use change (*N* ^a^ = 34)	16	26	14	17	15	13
Urban heat island (*N* ^a^ = 3)	2	1	–	1	1	1
Noise pollution (*N* ^a^ = 12)	5	8	5	6	4	4
Artificial light at night (*N* ^a^ = 1)	1	–	–	–	–	–
Food resources (*N* ^a^ = 11)	4	6	4	2	3	3
Land‐use change + Urban heat island (*N* ^a^ = 2)	2	1	–	–	–	–
Land‐use change + Artificial light at night (*N* ^a^ = 1)	1	1	–	–	–	1
Land‐use change + Food resources (*N* ^a^ = 11)	1	3	4	5	2	4
Land‐use change + Urban heat island + Food resources (*N* ^a^ = 3)	2	2	–	1	–	1
Land‐use change + Urban heat island + Noise pollution + Artificial light at night (*N* ^a^ = 1)	1	1	–	1	–	–
Urban heat island + Artificial light at night (*N* ^a^ = 1)	1	–	1	1	–	–
Total (*N* ^a^ = 80)	36	49	28	34	25	27

Abbreviation: –, Studies did not include that life‐history traits.

^a^ Number of studies under each environmental factor category.

^b^ Number of studies including life‐history traits under each environmental factor category.

### 2.4 A unified conceptual framework for assessing interactions among environmental factors and breeding fitness

Previous reviews have suggested several frameworks to assess the mechanisms and ecological consequences of noise and/or ALAN (Francis & Barber, 2013; Gaston et al., 2013; Swaddle et al., 2015), but we know little about how multidimensional environmental factors interact and shape fitness. Therefore, we propose a new conceptual framework that encompasses all four environmental factors and species fitness to guide future research, particularly on the interactions between food resources and other environmental factors (Figure 2). The interaction between land‐use change and UHI on species likely imposes a synergistic effect, as both can reduce food resources and disrupt breeding habitats (Opdam & Wascher, 2004; Sohl, 2014; Williams et al., 2022). In addition, noise pollution and ALAN can produce synergistic effects on population dynamics and breeding fitness by disrupting foraging, mating, communication, reproductive behaviors, and physiological responses (Dominoni, Halfwerk, et al., 2020; Kight & Swaddle, 2011; Lowry et al., 2013; Navara & Nelson, 2007; Senzaki et al., 2020). In the sections below, we aim to follow the framework (Figure 2) and describe through which mechanistic pathways (e.g., laying, mating, and communication behaviors, and physiological responses) urbanization determines the breeding performance for certain species in urban areas, leading to changes in life‐history traits and breeding fitness.

## 3 SPECIES RESPONSES TO URBANIZATION AND RELATED ENVIRONMENTAL FACTORS

**FIGURE 3:**
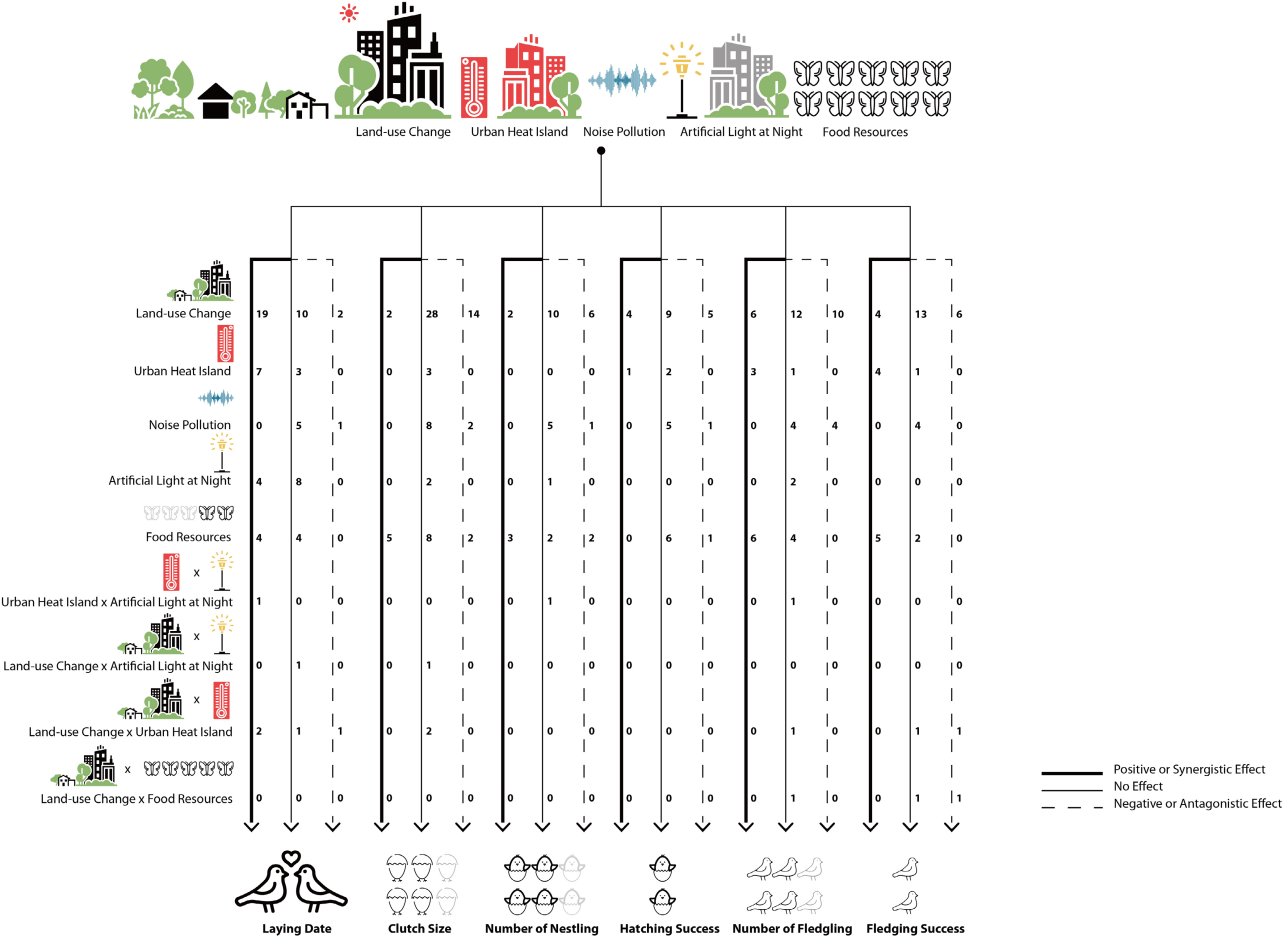
Diagram illustrating patterns and summaries from the results of systematic review about the effects of land‐use change, urban heat island (UHI), noise pollution, artificial light at night (ALAN), food resources, and any interactions between these factors, on life‐history traits and breeding fitness. For laying date, a “Positive Effect” indicates advanced laying dates, but does not imply that advanced laying dates produce positive effects on breeding fitness for the species. For the remaining life‐history traits, “Positive Effect” denotes better reproductive performance (larger clutch size, higher number of nestlings or fledglings, higher hatching, or fledgling success) in urban areas, or at higher temperature, noise, light illumination, or with more abundant food conditions. Numbers besides the arrows represent the number of times that studies report such an effect. As for combined effects of these environmental factors available from the systematic review, we compared the interactive effects between environmental factors to the individual effects reported in the studies—for example, comparing the interaction of UHI and ALAN to individual effects of UHI and ALAN—and then identified whether the interactive effect produced poorer or better effects on life‐history traits and breeding fitness. If the combined effect did more harm compared to individual effects, a synergistic effect was then documented. Likewise, if the combined effect offset the negative effects of the individual factors, the antagonistic effect was then documented. We did not find any positive effects produced by combined effects and thus do not visualize this possibility; however, we cannot rule out the possibility that environmental factors may produce positive effects and in combination the positive effects get enhanced in some cases. For laying date, if multiple factors led birds to advance their laying dates more, then it is denoted as synergistic effect. For the remaining life‐history traits, if birds had smaller clutch sizes, lower number of nestlings or fledglings, or lower hatching or fledging success due to the interaction of multiple factors, this is denoted as synergistic effect.

Species' responses to urbanization have been studied extensively (Alberti, 2015; Chamberlain et al., 2009; Sih et al., 2011). There are now empirical studies showing environmental factors linked to urbanization have direct effects on fitness of species (Ferraro et al., 2020; Zhao et al., 2022). Here, we used our framework to assess the separate and combined effects of environmental factors on species' life‐history traits and fitness, and particular attention was paid to how these interactions shape food resources (Figure 2). We then classified these effects as positive (i.e., greater reproductive outcomes), negative (poorer reproduction), or neutral responses (Figure 3; Acasuso‐Rivero et al., 2019; Ghalambor et al., 2007) in order to determine the cost of living in urban environments and in what instances species are actually benefiting from these theoretically disadvantageous environmental conditions.

### 3.1 Land‐use change effects on breeding fitness

Human‐driven land‐use change is one of the main forces driving the destruction of natural habitats (Fischer & Lindenmayer, 2007; Newbold et al., 2015). The intensity of habitat transformation can be categorized, based on the percentage of intactness of the original habitat, as habitat loss, fragmentation, or degradation (i.e., containing 0%–10%, 10%–60%, 60%–90%, and 90%–100% of the original habitat, respectively; Fischer & Lindenmayer, 2007). Habitat loss implies that species cannot access their preferred habitats, while habitat fragmentation and degradation usually result in patch‐isolation, elongated edge lengths, and massive loss of native vegetation (Haddad et al., 2015; Thompson et al., 2022). Consequently, breeding activities in these habitats can be compromised. Results from our systematic review on the impacts of land‐use change on key life‐history traits and breeding fitness show that 17 of 26 studies that studied the timing of breeding showed advances in laying dates linked to increasing urbanization, with this result found in five species; and 11 of 37 studies on six species have consistently reported larger clutch sizes in rural areas than urban or peri‐urban areas (Figure 3). There was no consistent pattern with regard to the number of nestlings (17 studies on 12 species) or fledglings (28 studies on 17 species), and hatching (18 studies on 13 species) or fledgling (21 studies on 13 species) success across species, with some species responding positively and others negatively to landscape urbanization.

#### 3.1.1 Negative responses to land‐use change

It is commonly assumed that land‐use change associated to urbanization leads to negative responses of species by promoting earlier laying dates and smaller clutch sizes (McDonnell & Hahs, 2015; Whitehouse et al., 2013). The costs of breeding earlier are usually associated with mismatches in peaks between the timing of breeding and the availability of food resources (Hajdasz et al., 2019; Visser & Gienapp, 2019). Overall, in urban and peri‐urban areas, carnivorous species (Wilman et al., 2014) like Crested Goshawks *Accipiter trivirgatus* (Lin et al., 2015) start breeding 8–33‐day earlier in man‐made structures in urban areas than in rural areas. Insectivorous and omnivorous species like Mountain Chickadees *Poecile gambeli* (Hajdasz et al., 2019; Marini et al., 2017) and Great and Blue Tits (Glądalski et al., 2015; Seress et al., 2018; Wawrzyniak et al., 2015) experience 2–14 days of advancement in laying dates when breeding in urban areas compared to nonurban areas, which is associated with mild temperatures and available anthropogenic food resources. Additionally, Mazumdar and Kumar (2014) suggested that smaller clutch sizes in urban areas may be the cost of dealing with the shortened length of food peaks in urban areas. Similarly, Bailly et al. (2016) suggested that while body condition and responses to local environment constraints are similar across habitats for some species (Great and Blue Tits), smaller clutch sizes could be a negative response to high nest failure and nestling mortality rates in urban areas. For instance, insectivorous species like Pied Flycatchers *Ficedula hypoleuca* (Vaugoyeau et al., 2016) produce 0.6 eggs fewer in urban environments than in rural and natural areas probably due to marked temperature fluctuations. Omnivorous species like Great and Blue Tits (Bailly et al., 2016), and Purple Sunbirds *Nectarinia asiatica* (Mazumdar & Kumar, 2014) have been reported to lay 0.5–3.6 eggs less in urban than nonurban areas due to unpredictable food resources and a harsh nesting microclimate.

#### 3.1.2 Positive responses to land‐use change

Some species show positive responses to urbanization, for instance, by having especially flexible diets and through niche expansion (Kark et al., 2007; Pagani‐Núñez et al., 2019). In some instances, this may result in increased breeding fitness. Urban areas can in fact provide multiple food resources to many different taxa facilitated by extensive urban greening. Green areas usually harbor a great diversity of urban trees that can result in multiple small food peaks (Haddad et al., 2015). In addition, residential areas can provide stable anthropogenic food, such as refuse, nuts, and sunflower seeds, and harbor large prey populations, which can benefit granivorous, omnivorous, and carnivorous species (Kark et al., 2007; McCabe et al., 2018; Robb et al., 2008). Multiple, alternative food resources in urban areas can enhance body condition of breeding females during the pre‐laying period (Harrison et al., 2010), which can favor egg formation by enabling females to produce more residual yolk and extra nutrients for offspring development (Marri & Richner, 2014). Therefore, abundant alternative food resources in urban areas seem to represent an advantage for species with traits that enable the exploitation of these resources. In return, these species are more likely reproduce over time and may achieve similar or higher fitness than conspecific populations in nonurban areas. Potential positive effects of urbanization linked to such responses are also apparent in the studies included in our systematic review. For example, granivorous species such as Eurasian Coots *Fulica atra* living near urban pond complexes can produce 0.5 more eggs in association with increased food resources in human‐maintained waterbodies (Minias, 2016). Omnivorous species such as European Blackbirds achieve 36% higher fledging success in urban than nonurban areas because of human presence potentially alleviating the amount of nest predation around their nesting environments in gardens and parks (Ibáñez‐Álamo & Soler, 2010). Carnivorous species like Eurasian Kestrels *Falco tinnunculus* (Sumasgutner et al., 2014) and Northern Goshawks (Solonen, 2008) achieve either higher hatching success (+10%–20%) or produce 0.3–2.7 more nestlings due to more stable food sources (i.e., small mammals and birds) in urban than in nonurban areas (Kettel et al., 2019; Suri et al., 2017). Interestingly, despite many studies linking widespread phenotypic changes as an adaptation to an urban lifestyle (Sepp et al., 2018), land‐use change (i.e. the destruction or transformation of natural and semi‐natural habitats) may not be the direct driver. The UHI effect, noise pollution, ALAN, and its effects on food resources may also be important factors driving this pattern (Dominoni et al., 2013; Seress et al., 2020; Visser & Gienapp, 2019).

### 3.2 Urban heat island effects on breeding fitness

The UHI effect has been linked to phenological changes, such as mismatches between the peaks of food resources and the timing of breeding, which may cause food shortages and result in reduced breeding fitness (Reed et al., 2013; Visser & Gienapp, 2019). Surprisingly, from the results of our systematic review, no consistent pattern was found since only 11 studies on eight species have reported the impact of temperature on key life‐history events and breeding fitness (Figure 3). Great and Blue Tits are the only two species found in seven of 11 studies that tend to advance their laying dates with increased spring temperature. Three studies on two species have reported no effect of spring temperature change on clutch size. There was no consistent pattern with regard to the number of nestlings (one study on one species) or fledglings (two studies on two species). Interestingly, hatching (one study on three species) and fledgling (two of three studies on four species) success was higher with increased temperatures in urban areas.

#### 3.2.1 Negative responses to the urban heat island effect

The UHI effect can result in negative responses from species by promoting earlier laying dates in particular for long‐distance migratory species for which it is difficult to predict weather conditions and food abundance thousands of kilometers away (Møller et al., 2008; Tuomainen & Candolin, 2011). Urban areas experience both higher daytime surface temperature and nighttime atmospheric temperature than surrounding areas on average (Peng et al., 2012). This is mostly due to urban‐built structures capturing solar radiation and decreasing convection efficiency as much as 58% (Zhao et al., 2014). In winter, the intensity of the UHI effect is higher in the most populated cities. Heat accumulated during the winter can result in disproportionally high temperatures during early spring (Oke, 1995), which can result in phenological mismatches between predators and their prey (Samplonius et al., 2020). However, no study included in our systematic review shows direct negative effects of the UHI effect on life‐history traits or breeding fitness of bird species. This suggests that species able to persist in urban environments generally are well equipped to cope with temperature changes associated with urbanization, or that the impact of UHI effect on life‐history traits except for laying date has been underappreciated.

#### 3.2.2 Positive responses to the urban heat island effect

The UHI effect can lead to positive responses of species by promoting earlier laying dates when warmer environments advance plant phenology simultaneously and the peak of food demand is synchronized with potential food resources (Crick, 2004; Hadfield & Reed, 2022; Samplonius et al., 2020). Increased temperatures during winter and early spring can trigger positive responses in some species, maintaining better body condition, advancing their laying dates, and reducing the likelihood of experiencing asynchrony with their preferred food sources (Burgess et al., 2018; Lehikoinen et al., 2006; Renner & Zohner, 2018; Rockwell et al., 2012). Indeed, it has been shown that the annual surface temperature across global cities has increased 1.5°C on average since 2003 (Peng et al., 2012), which has advanced up to 10 days the onset of flowering and leaf‐out phenology in hundreds of species (Wohlfahrt et al., 2019). This advancement of plant phenology has been linked to an earlier appearance of insects. For example, in experimental conditions, a 3°C temperature increase can advance 11 days the egg‐hatching activity of Oak Winter Moths *Operophtera brumata* and bud burst of European Oaks *Quercus robur* (Buse & Good, 1996). Additionally, increased temperatures can advance the timing and increase the availability period of certain food resources. For example, 1.97–2.97°C warmer temperatures than control conditions can extend the provision window of Tent Caterpillars *Malacosoma californicum pluviale* by 25 days (Kharouba et al., 2015). From the results of our systematic review, we found no consistent pattern with regard to the effect that increased temperature has on laying dates. However, there are many studies showing that UHI may have contributed to increase fledging success. For example, omnivorous species like Great and Blue Tits (Glądalski et al., 2015, 2016; Solonen & Hildén, 2014; Whitehouse et al., 2013) have been reported to advance their laying dates by 1.4–2.4 days for each degree Celsius increase. While one omnivorous species, Western Jackdaws *Corvus monedula* (Meyrier et al., 2017) showed little or no responses to high‐temperature conditions regarding hatching or fledging success, omnivorous species like American Robins *Turdus migratorius*, insectivorous species like Black‐headed Grosbeaks *Pheucticus melanocephalus*, granivorous species like Mourning Doves *Zenaida macroura* (Becker & Weisberg, 2015), and carnivorous species like Eurasian Kestrels (Kreiderits et al., 2016) achieve 10%–12% higher fledging success rate due to a temperature increase of 5–15°C in certain urban areas. While the effects of climate change on egg‐laying behaviors have received long‐lasting attention (Crick, 2004; Crick & Sparks, 1999; Sparks & Carey, 1995), few studies have explored the interactions between UHI and other environmental factors linked to urbanization and how they shape food resources, life‐history traits, and breeding fitness.

### 3.3 Noise pollution effects on breeding fitness

Noise pollution can change the acoustic environment triggering behavioral and physiological responses and lead to fitness consequences (Halfwerk & Jerem, 2021; Kight & Swaddle, 2011). Noise pollution caused by road traffic, construction works, and industrial factories can change different aspects of the acoustic environment, such as spectrum (frequency), intensity (loudness or amplitude), and duration (abruptness or chronicity) (Francis & Barber, 2013). Interestingly, our systematic review reveals some interesting patterns. Thirteen studies on nine species have reported the impact of noise pollution on key life‐history traits and breeding fitness (Figure 3). One of six studies found that one species tended to delay their laying dates with higher noise levels, but five studies showed little effect on laying dates. Two studies on two species reported smaller clutch sizes and seven studies on six species found no response in noisier habitats. Four of eight studies displayed a consistent pattern in that higher noise levels are negatively associated with the number of nestlings (one study on one species) and fledglings (four studies on four species) while the other four showed little effects. Hatching (five studies on five species) or fledgling (four studies on four species) success generally showed no consistent responses to noise except for one species experiencing reduced breeding success in noisy habitats.

#### 3.3.1 Negative responses to noise pollution

Noise pollution can elicit negative responses of species by delaying egg laying and causing smaller clutch sizes (Injaian, Poon, & Patricelli, 2018). A potential driver of this pattern is that noise pollution reduces pairing success (Habib et al., 2007) and impairs individual mating ability (Wong & Candolin, 2015). Consequently, mate encounter rate and pairing success might decrease or be delayed. A delay in pairing timing may directly influence egg‐laying date in noisy habitats. In addition, noise pollution masks the alarm calls of species and leads to decreased ability of predator detection and ultimately reduced foraging efficiency and a decreased in the availability of food resources for the offspring (Templeton et al., 2016; Tilgar et al., 2022). From our systematic review, only one study showed such a negative response, with an increase of 10.6 dBA noise measured at nest boxes delaying first egg‐laying dates of Tree Swallows *Tachycineta bicolor* are by 3.8 days (Injaian, Poon, & Patricelli, 2018). Insectivorous species like Tree Swallows (Injaian, Poon, & Patricelli, 2018) and omnivorous species like Great Tits (Halfwerk, Holleman, et al., 2011) have been reported to lay 10% fewer eggs when ambient noise levels increase by 10–20 dBA.

Noise pollution can also result in negative responses by reducing fledging success (Acasuso‐Rivero et al., 2019; Patricelli & Blickley, 2006). More specifically, parent‐nestling communication can be affected in noisy environments. Many insectivorous and omnivorous species are altricial birds, which means their hatchlings are born blind and therefore are only responsive to acoustic signals at the beginning of their post‐hatching development (Redondo & Reynai, 1988). Consequently, noise can mask acoustic signals produced by parents and thus decrease the intensity of nestlings begging, which may result in reduced provisioning and growth rates (Haff & Magrath, 2011; Lucass et al., 2016). Additionally, increased noise exposure can impair foraging activities and antipredator behaviors (Kight et al., 2012; Kight & Swaddle, 2011; Quinn et al., 2006), which can result in increased foraging time and lower prey encounter compared to quiet areas. These effects combined can lead to smaller brood sizes in noisy environments. The results of our systematic review provide empirical evidence that noise overlapping with birds' song frequencies can reduce the number of fledglings. For instance, environmental noise with frequency range between 1 and 5 kHz can strongly overlap with Eastern Bluebirds' song and thus led a decrease of two to three fledglings (Kight et al., 2012). Traffic noise with ambient levels of 40–50 dB in April had a negative effect on the number of Great Tit fledglings, while traffic noise with a 2 kHz frequency has been linked to reduced clutch size in this species (Halfwerk, Holleman, et al., 2011). Insectivorous species like flycatchers are also severely affected by noise. Ash‐throated Flycatchers *Myiarchus cinerascens* had two fewer fledglings than control groups when playback speakers increased noise at the next boxes by 20 dB from 43 dB (Mulholland et al., 2018). Likewise, Pied Flycatchers breeding within 20 m of roads had up to five fewer fledglings than pairs breeding 106 m away from roads near a boreal coniferous forest (Kuitunen et al., 2003).

#### 3.3.2 Positive responses to noise pollution

While the effects of noise pollution on species have been thoroughly investigated (Candolin & Wong, 2019; Francis & Barber, 2013), few studies have explored positive or ameliorated responses to noise. Noise pollution can lead to positive or no responses of species by building more resistance or promoting higher phenotypic plasticity (Halfwerk, Bot, et al., 2011; Slabbekoorn & Peet, 2003), which may indirectly mitigate potential negative effects on breeding fitness. In our systematic review, we recorded no positive effects of noise pollution, but several reports of little or no effects instead. Little or no effects of noise pollution on fitness could be the result of noise pollution offsetting negative effects of other environmental factors. More specifically, granivorous species like Zebra Finches (Potvin & MacDougall‐Shackleton, 2015) and House Sparrows (Meillere et al., 2015; Schroeder et al., 2012) show no response to noise generated by experimental recordings (~63 dB at the nests) or electricity generators (~68 dB) in terms of clutch size, hatching success, and number of fledglings. Authors implied that increased song amplitudes and frequencies of these species are likely adaptions to signal masking in high noise environments (Meillere et al., 2015; Potvin & MacDougall‐Shackleton, 2015). Similarly, insectivorous species such as Eastern, Western, and Mountain Bluebirds, Ash‐throated, and Pied Flycatchers, and Tree Swallows have been reported to display little or no effects on different life‐history traits in varied contexts in response to noise (Injaian, Poon, & Patricelli, 2018; Injaian, Taff, & Patricelli, 2018; Kight et al., 2012; Kleist et al., 2018; Kuitunen et al., 2003; Mulholland et al., 2018). Studies measuring oxidative stress levels suggest that these species might have developed a tolerance to noise despite a relative high level of stress compared to control groups (Injaian, Taff, & Patricelli, 2018; Kleist et al., 2018).

### 3.4 Artificial light at night effects on breeding fitness

ALAN can alter behavioral and physiological responses of species (Dunlap et al., 2003; Gaston et al., 2013) and lead to decreased breeding fitness (Dominoni, Halfwerk, et al., 2020). ALAN is mainly associated with human settlements and transportation networks and strongly varies in space and time (Gaston et al., 2013). From the results of our systematic review, four studies have reported the effect of ALAN on 11 avian species (Figure 3), yet no consistent pattern was found. For instance, four out of the 11 species tend to advance their laying dates, while eight species show little or no response to ALAN.

#### 3.4.1 Negative responses to artificial light at night

ALAN can lead to negative responses of species by promoting earlier laying dates via physiological mechanisms (Dunlap et al., 2003; Sanders et al., 2021). ALAN can disrupt individuals' circadian rhythms through the release of the hormone progesterone, suppressed melatonin secretion, and shortened sleeping time (Raap et al., 2015). These hormonal changes accelerate gonadal growth (Dominoni et al., 2013) and cause increased physiological stress (Dunlap et al., 2003). The premature development of reproductive glands may promote earlier egg‐laying behaviors, with three studies providing empirical evidence of this pattern in our systematic review. Specifically, omnivorous species such as Great Tits (Dominoni, Kjellberg Jensen, et al., 2020), Blue Tits (De Jong et al., 2018), and European Blackbirds (Russ et al., 2017) advance their laying date by 2.1–7 days compared to individuals breeding in areas with little ALAN. Despite the fact that a few studies have linked advanced laying behaviors with higher breeding fitness in urban and rural areas (Antonov & Atanasova, 2003; Mennechez & Clergeau, 2006), our systematic review did not uncover studies describing effects on breeding fitness linked to earlier laying dates triggered by ALAN.

#### 3.4.2 Positive responses to artificial light at night

Species can show positive responses to ALAN, such as improved fledging success (Dominoni, Halfwerk, et al., 2020; Senzaki et al., 2020). ALAN can provide extra foraging opportunities by extending the time available to find food (Sanders et al., 2021; Wang et al., 2021). Also, ultraviolet (UV) and LEDs lights can enhance the ability of the four‐photoreceptor pigments possessed by birds to detect prey under low‐light conditions (Gaston et al., 2013). Extended time of foraging can thus result in a higher amount of food being delivered to offspring and higher fledging success (Senzaki et al., 2020), or at least compensate for the lesser amounts of food that species can collect in urban areas during the daytime. An example of higher reproductive performance in elevated ALAN included in our systematic review was that of European Blackbirds, which can extend their foraging time up to 50 min in city centers compared to forest birds. Extended foraging time was the product of being exposed to 0.44 ± 0.36 lux (calculated based on the citywide lamp‐density map at study sites; Russ et al., 2015), which is linked to enhanced fledging success (Russ et al., 2017).

## 4 EFFECTS OF URBANIZATION AND RELATED ENVIRONMENTAL FACTORS ON FOOD RESOURCES AND TROPHIC INTERACTIONS

Food of avian species mainly consist of plant‐based resources, and of invertebrates and vertebrate animals (Pigot et al., 2020), and urbanization and related environmental factors can have direct and indirect effects on these trophic levels (Burgess et al., 2018). For example, urbanization has advanced the timing of leaf sprouting, flowering, and fruiting in urban areas (Wohlfahrt et al., 2019). This, in turn, can directly impact life‐history traits and breeding fitness of herbivorous, granivorous, nectarivorous, and frugivorous species due to increased asynchrony between key life‐history stages and peaks of food sources (Pigot et al., 2020; Renner & Zohner, 2018). Likewise, omnivorous, invertivorous, and vertivorous species will be influenced by population dynamics of species from lower trophic levels (Faeth et al., 2005; Pigot et al., 2020; Samplonius et al., 2020). Insectivorous species have experienced population declines of 13% in Europe (Bowler et al., 2019) and 31.8% in North America (Rosenberg et al., 2019), and such reduction has been attributed to the loss of insect diversity and biomass and other environmental factors (Dirzo et al., 2014). Traditionally, bottom‐up (producer‐driven) and top‐down (predator‐driven) regulation theories are the main approaches proposed to describe population dynamics across trophic levels (Abdala‐Roberts et al., 2019; Hunter & Price, 1992; Vidal & Murphy, 2018), but few studies have considered how environmental factors interfere with biotic interactions in urban environments (as argued by Shochat et al., 2006). Here, we used our novel framework (Figure 2) to analyze how urbanization and related environmental factors affect the trophic levels and shape food sources of avian species, to further ascertain the drivers of these disturbance patterns.

### 4.1 Land‐use change, food resources, and trophic interactions

Land‐use change can result in reduced primary productivity in urban areas (Imhoff et al., 2000), and this may directly limit species richness and abundance (Marzluff et al., 2001; Pickett et al., 2011). However, studies show that plant biomass may only have a marginal effect on the interaction between insectivorous birds and insects, and vice versa. For example, despite that plant biomass increases 61%–65% by fertilization, increased plant‐based food resources have little effects on the interaction between herbivores and predators from high trophic levels (Borer et al., 2006). This, therefore, suggests that, in urban areas, plant biomass may not be the only factor determining the interactions between insects and birds. In other words, when invertebrate communities are able to persist in urban environments, the abundance of herbivorous insects may be influenced by other factors. For example, land‐use change can limit the capacity of arthropods with low‐dispersal ability to recolonize new areas. Thus, this may lead to a reduction in population sizes and local extinctions due to the fact that increased impervious surfaces and fragmented habitats directly reduce habitat suitability or connectivity (Beninde et al., 2015; Fenoglio et al., 2020). More specifically, land‐use change as one of the drivers of global deforestation has contributed to population declines of 33% of insect species, with Orthoptera and Coleoptera being the most affected (Dirzo et al., 2014; Li et al., 2022). One long‐term study indicates that 62% of moth species (417 out of 673) experience a significant decline or have a tendency to do so due to habitat modifications (Fox et al., 2014), while 21 resident butterflies have gone locally extinct due to habitat conversion from meadows and grasslands to pasture and deciduous trees (Nilsson et al., 2008). This illustrates how land‐use changes have a tremendous impact on insects and could thus constrain their predators' populations.

While it seems that plant biomass has a relatively limited impact on the interaction between insects and birds, increased plant diversity and abundance in urban areas can have a direct positive impact on birds (Shochat et al., 2006). Land‐use change often implies enhanced management of urban green spaces (e.g., city parks/parklands, cemeteries, public golf courses, and university campuses), and these spaces can mitigate primary productivity loss (Antrop, 2004; Faeth et al., 2005; Pickett et al., 2011). In these areas, extended plant growing seasons and abundant fruit resources from urban trees can offer omnivorous bird species food resources, which helps them to cope with negative land‐use change effects. This is in line with the pattern of our systematic review, with 11 articles involving the study of land‐use change and food resources. Omnivorous species like Western Jackdaws (Meyrier et al., 2017) and carnivorous species like Eurasian Kestrels (Kübler et al., 2005) experience little difference in clutch size and number of fledgling when breeding either in urban or in rural and natural areas, and this may be largely due to available anthropogenic food resources.

However, not all omnivorous species can benefit from anthropogenic food resources, either because despite their relatively generalized diets, there are omnivorous species somewhat specialized in certain taxa (Pigot et al., 2020) or because individuals show great behavioral variability that can explain their tendency to exploit anthropogenic food resources (Griffin et al., 2022). For instance, omnivorous species like Great Tits depend on caterpillars mostly during the breeding season, and larger caterpillar biomass due to the presence of mature trees in natural areas can help them achieve 1.2–3.6 eggs and one to three more fledglings than their urban counterparts (Seress et al., 2018). However, reproductive success is context dependent if patches of native plant communities kept in old neighborhoods provide abundant caterpillars (Narango et al., 2018). This means that food types also play a key role in determining changes in life‐history traits and breeding fitness of different omnivorous species (Robb et al., 2008). Likewise, for some species, the amount of food resources is similar in urban and nonurban areas but breeding fitness can differ. For example, despite abundant food in urban areas, carnivorous species such as Northern Goshawks (Solonen et al., 2019), Eurasian Kestrels (Sumasgutner et al., 2014), and Peregrine Falcons *Falco peregrinus* (Kettel et al., 2019) often have more fledglings in nonurban than urban areas. This implies that changes in land use and food resources are not exclusive factors determining breeding fitness of species able to colonize urban environments and that in urban areas other environmental factors may have more importance than food resources for breeding fitness. Further, the effects of urbanization on breeding performance could also be explained by biological features of the species (e.g., diet, body size).

### 4.2 Urban heat island effect, food resources, and trophic interactions

The UHI effect can mediate the strength of trophic cascades between secondary consumers and plants (Renner & Zohner, 2018; White, 2008). Increased temperature in urban areas has been linked to advanced leaf‐out phenology and a lengthened season for vegetation growth (i.e., these resources would be available over a longer period of time in urban than in nonurban areas), and this could mitigate the negative impact on food availability derived from land‐use changes (Fu et al., 2015; Zhao et al., 2014). For example, 343 Chinese cities have experienced an advancement of 10.5 days of the start of leaf‐development season for every 1°C temperature increase during the spring (Jia et al., 2021). Additionally, plant growing season have expanded by 2.2–4.4 days in the Northern Hemisphere (Wang et al., 2019). Rising temperatures in urban areas can advance 7–10 days the flowering and fruiting phenology of plants (Wohlfahrt et al., 2019). Nonetheless, advanced plant phenology in urban areas may not trigger a similar response of herbivorous insects, granivorous, nectarivorous, and frugivorous bird species, due to consumers having lower sensitivity to temperature increases than primary producers and therefore having delayed responses to advanced timing of reproduction (Thackeray et al., 2016; White, 2008). Hence, such asynchrony can lead to a decrease in the available food resources for species from high trophic levels and thus exacerbate the relatively poor food conditions in urban areas compared with natural or rural areas (Faeth et al., 2005; Shochat et al., 2006).

Furthermore, the UHI effect can reduce insect abundance available for avian species in urban areas by enhancing physiological stress and jeopardizing the fitness of arthropods (Deutsch et al., 2008; Dirzo et al., 2014). Oscillation of climate parameters can produce spatiotemporal alterations of temperature extremes and marked fluctuations, which can push the thermal tolerance of ectotherms to their limits (Huey et al., 2012). For example, a model simulation of thermal sensitivity of 38 insect species indicates that the mean fitness consequences of rising temperatures could be devastating for species within low‐latitude ranges, and that they are likely to suffer a 20% decrease in fitness due to small thermal safety margins (Deutsch et al., 2008). We also found some empirical evidence from the results of our systematic review: two studies show that omnivorous species such as Great and Blue Tits can advance laying behaviors 1.4 days for every 1°C temperature increase in urban areas but not lead to smaller clutch size compared with their rural counterparts (Glądalski et al., 2015; Wawrzyniak et al., 2015). This implies that rising spring temperatures in urban areas are favoring species and populations with early phenologies since it can bridge the asynchrony between urban food peaks and breeding demands (Samplonius et al., 2020). However, omnivorous (e.g., Western Jackdaws, Meyrier et al., 2017) and carnivorous species (e.g., Eurasian Kestrels, Kreiderits et al., 2016) may not be affected by the UHI effect, since these species can exploit a variety of food resources in urban areas (Pigot et al., 2020; Robb et al., 2008). Nonetheless, more studies on other taxonomic groups (e.g., insectivorous species) would provide deeper insights on the variation in phenological responses to rising temperatures.

### 4.3 Noise pollution, food resources, and trophic interactions

Noise pollution can, on the one hand, interfere with predator–prey interactions by both altering prey and predator behavior and, on the other hand, negatively impact survival of primary producers due to reduced predation pressure on primary consumers (Abdala‐Roberts et al., 2019; Classen‐Rodríguez et al., 2021; Shannon et al., 2016). Studies show that noise pollution drives birds species that are less tolerant to noise away from urban environments (Francis et al., 2009). This suggests that noise pollution might, in return decrease predation risk and interspecific competition, thereby leading to lower reproductive costs in noisy environments (Francis et al., 2009; Lima, 2009). For example, Western Scrub‐jays *Aphelocoma californica* were found to be 32% less abundant in noisy environments than in control sites, and Black‐chinned Hummingbirds *Archilochus alexandri* and House Finches *Carpodacus mexicanus*, which are affected by scrub‐jay nest predation, consequently had higher nest success in noisy sites (Francis et al., 2009).

Likewise, noise pollution can reshape insect abundance disproportionally due to differences in tolerance to noise by different species, which can affect their avian predators (Classen‐Rodríguez et al., 2021). Experimental studies using compressor noise (ranging from ambient noise levels of 54.9–80.8 dBA) and river noise playbacks (ranging from 35.1 to 97 dBA) showed a mixed‐effect pattern to which some arthropod orders responded positively (e.g., Coleoptera and Hemiptera), others responded negatively (e.g., Araneae and Orthoptera), and some showed little or no response (e.g., Lepidoptera and Diptera) (Bunkley et al., 2017; Gomes et al., 2021). As a result, changes in insect distribution and abundance can benefit species with different diets. Moreover, noise pollution can reduce insect abundance in urban areas by disrupting reproductive behaviors of arthropods (Classen‐Rodríguez et al., 2021; Dominoni, Halfwerk, et al., 2020). Noise can mask signal perception and disturb the search of potential mates for reproduction, thereby leading to reduced pairing success, quality, and quantity of offspring (Balakrishnan, 2016). For example, noise playback can completely halt mating of American Leafhoppers *Scaphoideus titanus* (Mazzoni et al., 2009), whereas Bark Beetles (Coleoptera: Curculionidae) can produce 43% fewer eggs under a radio treatment compared to natural environments (Hofstetter et al., 2014).

Therefore, insectivorous and omnivorous species that breed in noisy environments could be directly affected by decreased insect abundance of certain orders. Furthermore, noise can affect the interaction between predatory and herbivorous insects and cause cascading effects on plants (Shochat et al., 2006). For example, experimental noise treatments (20 dBA higher compared with control groups) can significantly reduce plant biomass by affecting the predation rate by the secondary consumer Asian Lady Beetles *Harmonia axyridis* on the primary consumer Soybean Aphids *Aphis glycines* (Barton et al., 2018). Subsequently, reduced plant biomass could affect the availability of food resources for granivorous species. Unfortunately, from our systematic review, we found that this trophic interaction is largely unexplored, and more research is thus needed.

### 4.4 Artificial light at night, food resources, and trophic interactions

ALAN can have direct positive effects on primary producers by advancing or delaying their phenology (Meng et al., 2022). However, this positive effect can be weakened by disrupted plant–insect interactions (Giavi et al., 2021; Grubisic & van Grunsven, 2021). Similarly, ALAN has direct negative effects on population sizes of primary consumers (insects), which has been reported to cause cascading effects downward (plants) and upward (birds) (Owens et al., 2020). As a result, ALAN may have strong impacts on plants, whereas their combined impact may compensate each other, thereby mediating the strength of trophic cascades between secondary consumers and plants (Grubisic & van Grunsven, 2021; Kehoe et al., 2022). ALAN advances leaf‐out and delays leaf coloring phenologies by an average of 8.9 and 6.0 days, respectively, compared with areas without ALAN and under similar temperature conditions (Meng et al., 2022). However, plant growth over longer periods of time may not lead to greater plant biomass due to reduced plant–pollination interactions (Giavi et al., 2021). Specifically, LED street lamps reduce 62% of pollination visits by nocturnal insects after dark, and lower pollination success (Knop et al., 2017) could thus result in fewer flowers, fruits, and seeds for granivorous, nectarivorous, and frugivorous insect and bird species.

In addition, ALAN has been shown to reduce insect abundance, prey of insectivorous, omnivorous, and carnivorous species by altering their behaviors (Owens et al., 2020; Sanders et al., 2021). Insects are attracted to stationary light sources or vehicle headlights, leading to exhaustion, predation, or collapse, and ultimately death (Boyes et al., 2021). ALAN also enhances interspecific competition between diurnal and crepuscular insects, and consequently, affected species may need to postpone or extend foraging activities, which would then shrink suitable time for mating activities (Owens et al., 2020). For example, with increased light intensity (50–500 lux) near the experimental container, male Oriental Fruit Moths *Grapholita molesta* have been observed to spend less time performing mating displays (e.g., fanning‐ and crawling activity) (Li et al., 2019). Similarly, 60–90 lumen LEDs reduced the sex pheromone secreted by female Cabbage Moths *Mamestra brassicae* by up to 500 ng compared to control groups (Van Geffen et al., 2015). Both disruptions would lead to lower mating success and fewer offspring, and ultimately fewer food resources for avian species.

On the other hand, ALAN can provide greater top‐down pressure on insect populations from secondary to primary consumers (i.e., increased predation rates and reduced insect abundance) (Senzaki et al., 2020). For instance, ALAN leads to increased population sizes and nesting activities of insectivorous species (e.g., Cliff Swallow *Petrochelidon pyrrhonota*) and birds of prey (e.g., Peregrine Falcon), which decreases the abundance of terrestrial insects (i.e., Diptera) around illuminated areas; this implies that food resources for tertiary and secondary consumers can increase in areas with ALAN (Nankoo et al., 2019). ALAN also extends the foraging time of omnivorous and insectivorous species such as European Blackbirds and Barn Swallows *Hirundo rustica* due to increased food abundance near lighting sources (Russ et al., 2017; Wang et al., 2021). From our systematic review, we found no studies showing effects of ALAN on plant–insect–bird interactions. Therefore, long‐term research on population dynamics incorporating diverse plants, insects, and bird species over relatively large scales and in different habitats is necessary to further our knowledge of the interactive effects of ALAN on wildlife.

## 5 COMBINED EFFECTS OF ENVIRONMENTAL FACTORS AND FOOD RESOURCES ON BREEDING FITNESS

In the existing literature, the combined effects of environmental factors on biodiversity have mostly been studied between land use and climate change (UHI; Mantyka‐Pringle et al., 2012), and between noise pollution and ALAN (Halfwerk & Jerem, 2021; Wilson et al., 2021). Food resources are usually investigated in studies about trophic interactions, yet, the specific effects of these factors on life‐history traits and breeding fitness are generally not considered (Holt & Comizzoli, 2022; Renner & Zohner, 2018). To the best of our knowledge, there are no studies that have directly investigated the interactions between all these factors and breeding fitness; this review aims to point out this crucial gap of knowledge.

### 5.1 Interactive effects of land‐use change and urban heat island on breeding fitness

Land‐use change and the UHI effect can have independent impacts on species (Figure 2), and their interaction could generate either synergistic or antagonistic effects (Galic et al., 2018; Williams et al., 2022). For example, in a model simulation of 50 species' breeding ranges, climate change was projected to reduce more than 50% of species' suitable ranges, whereas land‐use change was estimated to make 20% of these species' breeding ranges less suitable (Sohl, 2014). In comparison, the interaction between land use and climate change either mitigated the reduced breeding range effect (20 species) or slightly expanded the unsuitable range (eight species, Sohl, 2014). Consequently, reduced species' ranges may directly threaten breeding and population viability of species inhabiting the affected areas. The interactive effect of land‐use change and the UHI effect could be synergistic when habitat loss and fragmentation are greatest as well as when extreme‐hot weather events take place in urban areas (Mantyka‐Pringle et al., 2012). When these synergetic effects exceed species' thermal limits or strongly affect demographic rates (Selwood et al., 2015), it could lead to species' extirpations from urban environments. On the other hand, the interactive effect of land‐use change and UHI effect could also be positive when species are more resilient, have high tolerance to increased temperatures, or are able to adapt to human habitats (Galic et al., 2018; Mantyka‐Pringle et al., 2012; Travis, 2003). For example, some species become more abundant in northern than in southern cities due to the UHI effect, which increases local temperatures to match their optimal thermal limits, and other species that have flexible diets and broader niches are also able to breed or colonize new urban habitats (Kark et al., 2007; Pagani‐Núñez et al., 2019; Williams et al., 2022).

From the results of our systematic review, we found both synergistic and antagonistic effects in three studies. Two studies suggest that the interaction between land‐use change and temperature produces cumulative effects on the laying date of omnivorous species such as Great and Blue Tits, with birds either delaying or advancing reproduction in response to cold or heat waves more strongly in urban than nearby natural areas (Glądalski et al., 2015; Whitehouse et al., 2013). Interestingly, despite that the laying behavior of Great and Blue Tits differs in urban and rural areas, two studies reveal that not much difference has been found in term of the clutch sizes (Glądalski et al., 2015; Whitehouse et al., 2013). This suggests that some species are highly sensitive to exacerbating interactive effects, for which the capacity to show plastic responses seems crucial to maintain these populations (Capilla‐Lasheras et al., 2022). For number of fledglings, nonsignificant antagonistic effects have been recorded for these species, with, for example, urban Great Tits producing 1.5 fewer fledglings than in urban than in natural areas (Wawrzyniak et al., 2020). The differences in the number of fledglings may arise from differences in caterpillar abundance between urban and natural areas, although surprisingly several studies suggest that increased temperature has limited impacts on food abundance (Selwood et al., 2015; Seress et al., 2020).

### 5.2 Interactive effects of noise pollution and artificial light at night on breeding fitness

Noise pollution and ALAN can also have independent impacts and produce cumulative effects on species' populations and breeding fitness (Figure 2; Côté et al., 2016; Galic et al., 2018). For example, 56 out of 140 avian species showed population declines due to noise pollution, while ALAN is associated with population changes in another 13 species (Wilson et al., 2021). However, up to 20 species showed population declines due to synergistic effects of noise pollution and ALAN, whereas five species showed antagonistic effects (Wilson et al., 2021). Additionally, studies have shown that noise pollution and ALAN may influence habitat choice and thereby breeding fitness and success (Dominoni, Halfwerk, et al., 2020; Swaddle et al., 2015). Noise pollution is usually linked to negative effects on species' breeding fitness (Habib et al., 2007; Kight et al., 2012; Schroeder et al., 2012), whereas ALAN has been associated with increased breeding fitness (Russ et al., 2017; Senzaki et al., 2020; Wang et al., 2021). Therefore, unlike the synergistic interactions between land‐use change and UHI, the presence of noise pollution and ALAN could be antagonistic (Galic et al., 2018). When noise and light treatments are presented, Western Bluebirds in noise‐and‐light‐treatment groups had one more fledgling than in the light‐only‐treatment groups (Ferraro et al., 2020). In other words, although the existence of ALAN in this context might have detrimental effects on fledglings, when combined with noise pollution, the overall effect can be marginal. The observed antagonistic effect may arise from reduced predation risks and increased foraging time (Dominoni, Smit, et al., 2020; Francis et al., 2009; Lima, 2009). From our systematic review, we found no empirical studies investigating the interactive effects of noise pollution and ALAN on laying behaviors and breeding fitness. However, one recent study demonstrated that interactive effects of nocturnal noise and diurnal light, but not ALAN, triggered advanced laying behavior and caused smaller clutch sizes of Barn Swallows, whereas there were no interactive effects of noise and ALAN on breeding success (Zhao et al., 2022). This result may not be applicable to other species, since the Barn Swallow is a human commensal (Smith et al., 2018) and might be particularly well adapted to life in urban areas. Thus, more research on the separate and combined effects of noise pollution and ALAN on breeding fitness of non‐commensal species is urgently needed.

### 5.3 Cumulative effects of environmental factors and food resources on breeding fitness

Land‐use change and food availability are key factors shaping breeding fitness (Figure 2; Opdam & Wascher, 2004; Selwood et al., 2015; White, 2008). Habitat loss, fragmentation, and degradation strongly affect the abundance of food resources, suggesting that the discontinuity of original habitats and altered vegetation composition could produce cumulative effects and filter many species (Beninde et al., 2015; Fox et al., 2014; Nilsson et al., 2008). Similarly, UHI may mitigate the impact of land‐use changes at local scales and provide suitable breeding conditions by shifting the environment to be more favorable for species' thermal limits and by extending the time window in which food resources are available in urban areas. As a result, the interaction between UHI and land‐use change could become antagonistic (Mantyka‐Pringle et al., 2012; Sprau et al., 2017; Travis, 2003; Zhao et al., 2014). Noise pollution, ALAN, and food resources could also be factors that can sometimes have severe synergistic effects, and indirectly affect life‐history traits and breeding fitness, when food resources are scarce, noise pollution reduces pairing success and parent‐nestling communication efficiency, and ALAN disrupts circadian rhythms and gonadal growth in urban areas (Dominoni et al., 2013; Dunlap et al., 2003; Swaddle et al., 2015). On the other hand, the interactions between noise pollution, ALAN, and food resources could be synergistically positive due to increased food resources caused by UHI, especially in urban environments, particularly if noise pollution reduces predation risk and ALAN extends species' foraging time (Figure 2; Dominoni, Smit, et al., 2020; Lima, 2009; Wang et al., 2021). Overall, UHI and ALAN often elicit mixed responses, while noise generally has a negative impact on species. We acknowledge that these interactions and effects are complex and still rather inconsistent. Further research is needed to elaborate a more comprehensive map of these processes—we hope this study is a step further in this direction.

## 6 RESEARCH RECOMMENDATIONS

Our review proposes a multidimensional framework (Figures 2) and elaborates on the complex interactions among land‐use change and related environmental factors and their effects on key life‐history traits and breeding fitness of avian species. While we have focused on birds, as they are commonly seen as good proxies of overall biodiversity (Kati et al., 2004), we believe this framework can be easily applied to other vertebrate and invertebrate taxa. Such research can aid scientists, practitioners, and policymakers in predicting species' behavioral and physiological responses, understanding the consequence of urbanization for population dynamics, and implementing targeted mitigation measures. Further research in several directions below will provide a broader picture and ascertain mechanistic pathways by which environmental filtering acts on species.

### 6.1 Expanding taxonomic and geographic representation

From the results of our systematic review, we found that research on the effects of urbanization and environmental factors on avian systems has concentrated on *Parus* sspp., *Passer* sspp., and *Falco* sspp., which represented 46% of the selected studies. Another 41 species accounted for 54% of the selected studies. Therefore, conducting more research on those less studied species, such as herbivorous, granivorous, nectarivorous, and frugivorous species, would be extremely profitable. Studies on single or multiple environmental factors on multiple species from three trophic levels should merit special attention. Ninety percentage of the selected studies were carried out either in Europe or North America. More detailed investigations on a broader range of geographic locations in Asia, Africa, and South America are crucial to properly characterize the effects of urbanization on species' ecology and evolution (Mantyka‐Pringle et al., 2012; Sanders et al., 2021), since these three continents are now experiencing an intense urbanization process (United Nations, 2019). Furthermore, it would be useful to carry out research on birds and other taxa living in freshwater and marine ecosystems using the framework proposed here, as these diverse contexts may lead to different conclusions regarding the relative importance and cumulative effects of these environmental factors.

### 6.2 Creating standardized protocols

From the results of our systematic review, the impact of urbanization and related environmental factors on breeding fitness sometimes displays inconsistent results. These inconsistencies could be due to spatial and temporal variation in the effects of urbanization on biological traits, but in addition, they could be due to different protocols or data recorders being used, or to divergent methods employed to quantify urbanization. For example, Blue Tits have been reported not only to advance laying date in urban areas but also to show little responses even within Europe (Glądalski et al., 2015; Pollock et al., 2017; Vaugoyeau et al., 2016). Similar patterns can be found for other environmental factors (Sprau et al., 2017 vs. Dominoni, Smit, et al., 2020). Therefore, it would be greatly useful to produce standard protocols for documenting breeding parameters, land‐use change, UHI, noise pollution, ALAN, and food resources (Hardisty, 2013; Khalil et al., 2022). Our review is a starting point to develop and implement standard protocols to investigate the complex interactions that may otherwise not be possible to generalize using inconsistent methodologies. Additionally, using model species is interesting as it enables researchers to generalize patterns over large geographical areas, but this may also hamper our understanding of these processes, because different species and taxa from different regions can show divergent responses to urbanization and related environmental factors.

### 6.3 Performing more experimental research

From the results of our systematic review, we determined that only 25% of studies (*N* = 20) were experimental, and 90% of that focused on noise pollution and food resources (*N* = 18). Experimental methods are particularly useful because the different environmental factors linked to urbanization are usually correlated. It would be highly profitable to carry out experimental research testing the individual and combined impact of ALAN and temperature on breeding fitness of urban birds. Simultaneously studying how multiple environmental factors determine life‐history evolution and breeding success is slowly gaining momentum (Sprau et al., 2017; Zhao et al., 2022). So far, it remains unclear to what extent food availability is the main determinant of breeding success of birds (Seress et al., 2020), or to what extent changes in urbanization‐related environmental factors mediate this process. It would also be important to replicate these experimental approaches on other species and regions (Pollock et al., 2017). Noise pollution has been reported to produce mixed effects on breeding fitness, as shown in Section 3.3 (Kleist et al., 2018; Mulholland et al., 2018; Potvin & MacDougall‐Shackleton, 2015). Future research must utilize consistent methodologies in an attempt to reach scientific consensus.

### 6.4 Developing insights from the perspective of adaptation and evolution

Although there has been increasing interest on eco‐evolutionary dynamics and adaptive evolution in urban ecosystems (Alberti, 2015; Donihue & Lambert, 2015), clear evidence of the environmental factors driving evolutionary adaptions in urban areas is rather scarce. Studies have attempted to conceptualize the urban environment as an ecological or evolutionary trap, suggesting that urban environments may act as a sink for species (Acasuso‐Rivero et al., 2019; Battin, 2004). For instance, species' responses such as smaller clutch sizes in urban areas may be a plastic adaptation to deal with shortened length of food peaks and high nest failure or nestling mortality rates (Bailly et al., 2016; Mazumdar & Kumar, 2014). Furthermore, recent studies suggest that urban populations may have a greater ability to express phenotypic variation and genetic diversity than their nonurban counterparts (Capilla‐Lasheras et al., 2022; Thompson et al., 2022). Hence, more efforts should be devoted to distinguishing plastic traits from adaptive traits in urban environments (Capilla‐Lasheras et al., 2022; Donihue & Lambert, 2015).

### 6.5 Evaluating measures for mitigation

From the results of our systematic review, we found several studies that consider more than two environmental factors when studying their effects on life‐history traits and breeding fitness (Table 3). We only recorded synergistic effects between UHI and ALAN and between land‐use change and UHI on laying dates. However, it seems that advanced laying dates did not contribute to smaller clutch sizes or reduced number of nestling and fledglings. Therefore, more research is necessary to identify in what contexts (taxonomic groups and regions) the environmental factors studied here act synergistically to decrease breeding fitness. Having determined how the different environmental factors interact to affect life‐history traits and breeding fitness, proper planning strategies to mitigate the negative impacts of environmental factors and guaranteeing population viability of urban populations could be employed to enhance urban biodiversity (Dominoni, Halfwerk, et al., 2020). Based on the multidimensional framework developed here (Figure 2), a combination of measures mitigating the synergistic effects from land‐use change and UHI can reach greater effects than individual measures. For example, green corridors have been proposed to increase connectivity between fragmented habitats, which can increase food resources for species occupying small habitat patches (Beninde et al., 2015), and green infrastructure has also been employed to reduce UHI effects, favoring species near their thermal limits (Norton et al., 2015). Furthermore, creating vegetated artificial barriers or dense shrub hedges alongside traffic networks, and reducing blue wavelengths and intensity of lighting sources in urban areas have been reported to be effective countermeasures against the negative consequences of noise pollution and ALAN (Dominoni, Halfwerk, et al., 2020; Jägerbrand & Bouroussis, 2021; Swaddle et al., 2015). It would also be useful to evaluate how effective these measures are across time and assess the possibility of their systematic and large‐scale application in urban areas.

## 7 CONCLUSIONS

1. Our systematic review has provided evidence that land‐use change and related environmental factors can affect laying date, clutch size, breeding fitness, and success of species via altered laying, foraging, communicating behaviors, and abnormal physiological responses. Food resources are also affected by land‐use change and related environmental factors, and play a crucial role in the reproduction of vertebrates and entire trophic webs.
2. Land‐use change is a factor that can determine whether a species can persist, and it has dominant impacts on species, more than UHI, noise pollution, and ALAN have at a local scale. UHI and ALAN usually produce mixed responses from species, whereas noise pollution is generally linked with negative effects.
3. The interactions between land‐use change and related environmental factors on breeding fitness can be antagonistic to each other, and consequently, all factors may have little impact. Or the cumulative effect of land‐use change, UHI, and food resources can be synergistic and have a stronger impact collectively than they do independently. The cumulative effect of noise pollution and ALAN can offset the separate negative impacts of these factors on species in some cases.
4. We believe that the unified multidimensional framework proposed here can encourage new research directions, expanding taxonomic and geographic sampling, and create a unified approach for field or experimental research.
5. Assessing conservation strategies for mitigation can assist scientists, urban managers, and policymakers, thereby creating a more cohesive and sustainable community of practice, in order to build biodiversity‐friendly cities.

## AUTHOR CONTRIBUTIONS

**Sihao Chen:** Conceptualization (equal); data curation (lead); formal analysis (lead); methodology (equal); writing – original draft (lead); writing – review and editing (equal). **Yu Liu:** Data curation (equal); formal analysis (equal); writing – original draft (equal); writing – review and editing (equal). **Samantha C. Patrick:** Conceptualization (equal); supervision (equal); writing – original draft (equal); writing – review and editing (equal). **Eben Goodale:** Conceptualization (equal); supervision (equal); writing – original draft (equal); writing – review and editing (equal). **Rebecca J. Safran:** Conceptualization (equal); funding acquisition (equal); supervision (equal); writing – original draft (equal); writing – review and editing (equal). **Emilio Pagani‐Núñez:** Conceptualization (equal); methodology (equal); supervision (equal); writing – original draft (equal); writing – review and editing (equal).

### OPEN RESEARCH BADGES

This article has earned Open Materials and Preregistered Research Design badges. Materials and the preregistered design and analysis plan are available at [https://doi.org/10.6084/m9.figshare.23276912.v1].

## Supporting information


Appendix S1.

Appendix S2.

Appendix S3.

Appendix S4.

## Data Availability

Data are permanently archived in Figshare repository: Chen et al. (2023).
