# Supplementary material for: A multidimensional framework to quantify the effects of urbanization on avian breeding fitness
Source: Ecol Evol. 2023 Jul 3;13(7):e10259. doi: 10.1002/ece3.10259 (PMC10316489; doi:10.1002/ece3.10259)

A multidimensional framework to quantify the effects of urbanization on avian breeding fitness

Sihao Chen, Yu Liu, Samantha C. Patrick, Eben Goodale, Rebecca J. Safran, Emilio Pagani-Núñez

Article for Ecology and Evolution

Appendix S1

This appendix includes a flow chart showing the systematic review methods and results.

Figure S1. A flow chart showing the systematic review methods and results.


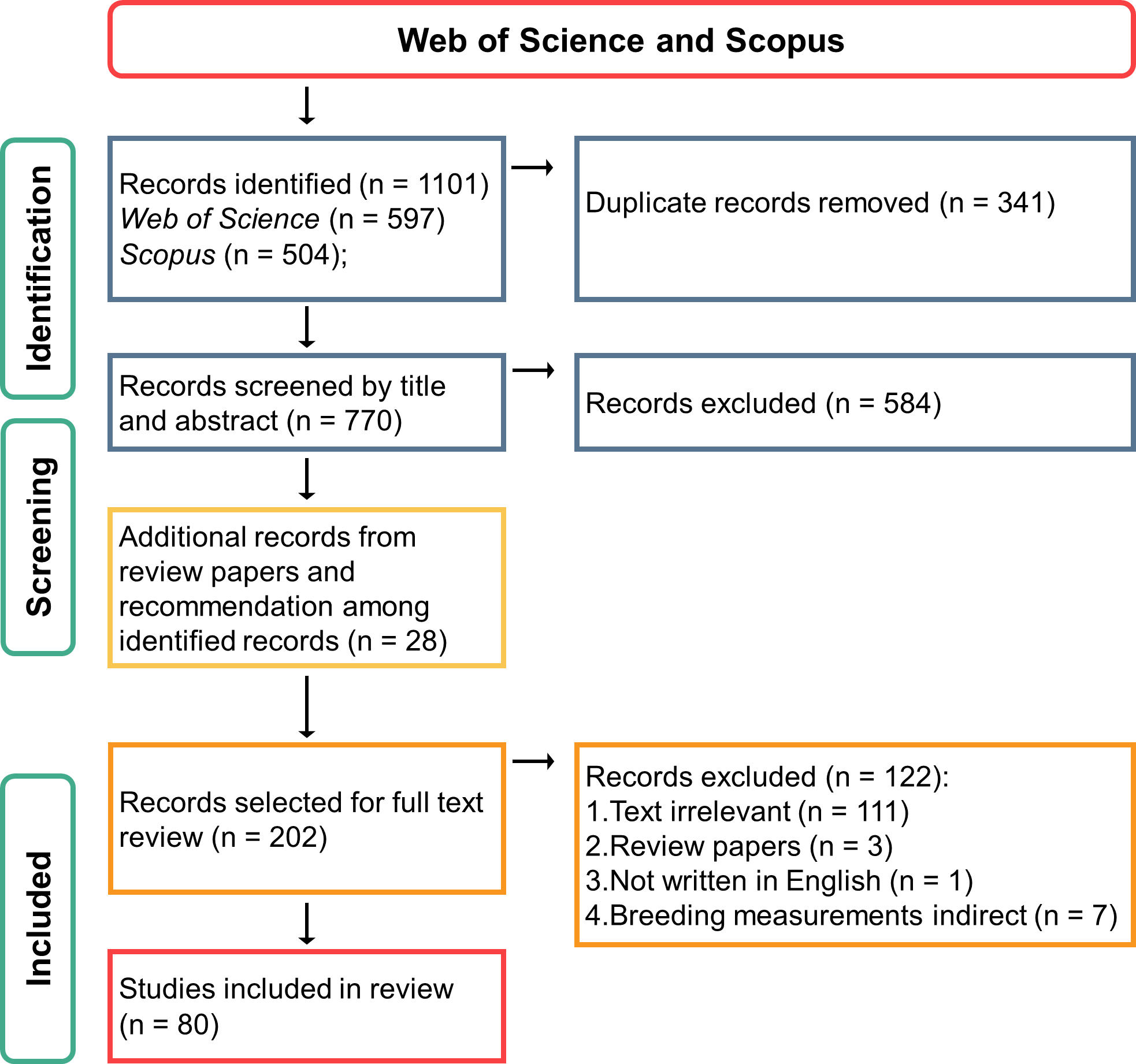

Supplement: Supplementary file 1 — Appendix S1. [file ECE3-13-e10259-s001.docx]
