# Supplementary material for: A multidimensional framework to quantify the effects of urbanization on avian breeding fitness
Source: Ecol Evol. 2023 Jul 3;13(7):e10259. doi: 10.1002/ece3.10259 (PMC10316489; doi:10.1002/ece3.10259)
Supplement: Supplementary file 2 — Appendix S2. [file ECE3-13-e10259-s004.docx]

A multidimensional framework to quantify the effects of urbanization on avian breeding fitness

Sihao Chen, Yu Liu, Samantha C. Patrick, Eben Goodale, Rebecca J. Safran, Emilio Pagani-Núñez

Article for Ecology and Evolution

Appendix S2

This appendix includes detailed information about three land-use change categories (i.e., urban, suburban and natural or rural areas), definitions of these categories, selected papers met these definitions.

Table S1. Studies using urban heat island as an environmental factor

| Term | Definition | Papers |
| --- | --- | --- |
| Natural / Rural (*N=49)* | With low or no proportion of built surfaces (<20% within study area), e.g., deciduous/coniferous forests, riparian forests, mixed woodland, national/ wilderness parks, grasslands, agricultural land, and farmland | (Solonen 2001; 2008; Rollinson and Jones 2002; Antonov and Atanasova 2003; Liven-Schulman et al. 2004; Millsap et al. 2004; Kübler, Kupko, and Zeller 2005; Mennechez and Clergeau 2006; Conway et al. 2006; Charter et al. 2007; Kelleher and O’Halloran 2007; Solonen and Ursin 2008; Berardelli, Desmond, and Murray 2010; Ibáñez-Álamo and Soler 2010; Frey et al. 2011; Seress et al. 2012; 2018; 2020; Hedblom and Söderström 2012; Cardilini et al. 2013; Whitehouse et al. 2013; Mazumdar and Kumar 2014; Solonen and Hildén 2014; Aldredge et al. 2014; Sumasgutner et al. 2014; Glądalski et al. 2015; 2017; Lin et al. 2015; Jarosław Wawrzyniak et al. 2015; J. Wawrzyniak et al. 2020; Bailly et al. 2016; 2017; Minias 2016; Vaugoyeau et al. 2016; Meyrier et al. 2017; Russ, Lučeničová, and Klenke 2017; Sprau, Mouchet, and Dingemanse 2017; Pollock et al. 2017; Biard et al. 2017; Thornton, Todd, and Roos 2017; Marini et al. 2017; Welch-Acosta, Skipper, and Boal 2019; Kettel et al. 2019; Solonen, Lokki, and Sulkava 2019; Hajdasz et al. 2019; Jarrett et al. 2020; Merling De Chapa et al. 2020; Najmanová and Adamík 2009; Charmantier et al. 2017) |
| Peri-urban (Suburban) (*N=6)* | With medium proportion of built surfaces (20-50% within study area), e.g., outskirts, a built-up area on the periphery of the city/town with open pastures, recreational facilities, and scattered buildings | (Rollinson and Jones 2002; Antonov and Atanasova 2003; Cardilini et al. 2013; Whitehouse et al. 2013; Aldredge et al. 2014; Thornton, Todd, and Roos 2017) |
| Urban (*N=45)* | With high proportion of built surfaces (>50% within study area), e.g., urban/town centers, residences, offices, commercial and industrial land, community, city parks/parklands, cemeteries, public golf courses, and university campuses | (Solonen 2001; 2008; Liven-Schulman et al. 2004; Millsap et al. 2004; Kübler, Kupko, and Zeller 2005; Mennechez and Clergeau 2006; Conway et al. 2006; Charter et al. 2007; Kelleher and O’Halloran 2007; Solonen and Ursin 2008; Berardelli, Desmond, and Murray 2010; Ibáñez-Álamo and Soler 2010; Frey et al. 2011; Seress et al. 2012; 2018; 2020; Hedblom and Söderström 2012; Mazumdar and Kumar 2014; Solonen and Hildén 2014; Sumasgutner et al. 2014; Glądalski et al. 2015; 2017; Lin et al. 2015; Jarosław Wawrzyniak et al. 2015; J. Wawrzyniak et al. 2020; Bailly et al. 2016; 2017; Minias 2016; Vaugoyeau et al. 2016; Meyrier et al. 2017; Russ, Lučeničová, and Klenke 2017; Sprau, Mouchet, and Dingemanse 2017; Teglhøj 2017; Pollock et al. 2017; Biard et al. 2017; Marini et al. 2017; Buxton, Santymire, and Benson 2018; Welch-Acosta, Skipper, and Boal 2019; Kettel et al. 2019; Solonen, Lokki, and Sulkava 2019; Hajdasz et al. 2019; Jarrett et al. 2020; Najmanová and Adamík 2009; Merling De Chapa et al. 2020; Charmantier et al. 2017) |

LITERATURE CITED

Aldredge, Robert A., Raoul K. Boughton, Michelle A. Rensel, Stephan J. Schoech, and Reed Bowman. 2014. “Hatching Asynchrony That Maintains Egg Viability Also Reduces Brood Reduction in a Subtropical Bird.” *Oecologia* 174 (1): 77–85. https://doi.org/10.1007/s00442-013-2749-x.

Antonov, Anton, and Dimitrinka Atanasova. 2003. “Small-Scale Differences in the Breeding Ecol Ogy of Urban and Rural Magpies Pica Pica.” *Ornis Fennica* 80: 21–23.

Bailly, Juliette, Bruno Faivre, Nadine Bernard, Mickaël Sage, Nadia Crini, Vincent Driget, Stéphane Garnier, Dominique Rieffel, and Renaud Scheifler. 2017. “Multi-Element Analysis of Blood Samples in a Passerine Species: Excesses and Deficiencies of Trace Elements in an Urbanization Study.” *Frontiers in Ecology and Evolution* 5: 1–9. https://doi.org/10.3389/fevo.2017.00006.

Bailly, Juliette, Renaud Scheifler, Sarah Berthe, Valérie-Anne Clément-Demange, Matthieu Leblond, Baptiste Pasteur, and Bruno Faivre. 2016. “From Eggs to Fledging: Negative Impact of Urban Habitat on Reproduction in Two Tit Species.” *Journal of Ornithology* 157 (2): 377–92. https://doi.org/10.1007/s10336-015-1293-3.

Berardelli, Daniele, Martha J. Desmond, and Leigh Murray. 2010. “Reproductive Success of Burrowing Owls in Urban and Grassland Habitats in Southern New Mexico.” *The Wilson Journal of Ornithology* 122 (1): 51–59. https://doi.org/10.1676/09-034.1.

Biard, Clotilde, François Brischoux, Alizée Meillère, Bruno Michaud, Manon Nivière, Stéphanie Ruault, Marie Vaugoyeau, and Frédéric Angelier. 2017. “Growing in Cities: An Urban Penalty for Wild Birds? A Study of Phenotypic Differences between Urban and Rural Great Tit Chicks (Parus Major).” *Frontiers in Ecology and Evolution* 5 (July): 1–14. https://doi.org/10.3389/fevo.2017.00079.

Buxton, Valerie L., Rachel M. Santymire, and Thomas J. Benson. 2018. “Mixed Effects of Urbanization on Density, Nest Survival, and Nestling Corticosterone of a Generalist Passerine.” *Ecosphere* 9 (12): e02517. https://doi.org/10.1002/ecs2.2517.

Cardilini, Adam P. A., Michael A. Weston, Dale G. Nimmo, Peter Dann, and Craig D. H. Sherman. 2013. “Surviving in Sprawling Suburbs: Suburban Environments Represent High Quality Breeding Habitat for a Widespread Shorebird.” *Landscape and Urban Planning* 115 (July): 72–80. https://doi.org/10.1016/j.landurbplan.2013.04.001.

Charmantier, Anne, Virginie Demeyrier, Marcel Lambrechts, Samuel Perret, and Arnaud Grégoire. 2017. “Urbanization Is Associated with Divergence in Pace-of-Life in Great Tits.” *Frontiers in Ecology and Evolution* 5 (May): 53. https://doi.org/10.3389/fevo.2017.00053.

Charter, Motti, Ido Izhaki, Amos Bouskila, and Yossi Leshem. 2007. “Breeding Success of the Eurasian Kestrel (Falco Tinnunculus) Nesting on Buildings in Israel.” Edited by Vincenzo Penteriani. *Journal of Raptor Research* 41 (2): 139–43. https://doi.org/10.3356/0892-1016(2007)41[139:BSOTEK]2.0.CO;2.

Conway, Courtney J., Victoria Garcia, Matthew D. Smith, Lisa A. Ellis, and Joyce L. Whitney. 2006. “Comparative Demography of Burrowing Owls in Agricultural and Urban Landscapes in Southeastern Washington: Burrowing Owl Demography.” *Journal of Field Ornithology* 77 (3): 280–90. https://doi.org/10.1111/j.1557-9263.2006.00054.x.

Frey, Caroline, Caroline Sonnay, Amélie Dreiss, and Alexandre Roulin. 2011. “Habitat, Breeding Performance, Diet and Individual Age in Swiss Barn Owls (Tyto Alba).” *Journal of Ornithology* 152 (2): 279–90. https://doi.org/10.1007/s10336-010-0579-8.

Glądalski, Michał, Miroslawa Banbura, Adam Kalinski, Marcin Markowski, Joanna Skwarska, Jaroslaw Wawrzyniak, Piotr Zielinski, Iwona Cyzewska, and Jerzy Banbura. 2015. “Inter-Annual and Inter-Habitat Variation in Breeding Performance of Blue Tits (Cyanistes Caeruleus) in Central Poland.” *Ornis Fennica* 92: 34–42.

Glądalski, Michał, Mirosława Bańbura, Adam Kaliński, Marcin Markowski, Joanna Skwarska, Jarosław Wawrzyniak, Piotr Zieliński, Iwona Cyżewska, and Jerzy Bańbura. 2017. “Differences in the Breeding Success of Blue Tits *Cyanistes Caeruleus* between a Forest and an Urban Area: A Long-Term Study.” *Acta Ornithologica* 52 (1): 59–68. https://doi.org/10.3161/00016454AO2017.52.1.006.

Hajdasz, Adrianne C., Ken A. Otter, Lyn K. Baldwin, and Matthew W. Reudink. 2019. “Caterpillar Phenology Predicts Differences in Timing of Mountain Chickadee Breeding in Urban and Rural Habitats.” *Urban Ecosystems* 22 (6): 1113–22. https://doi.org/10.1007/s11252-019-00884-4.

Hedblom, Marcus, and Bo Söderström. 2012. “Effects of Urban Matrix on Reproductive Performance of Great Tit (Parus Major) in Urban Woodlands.” *Urban Ecosystems* 15 (1): 167–80. https://doi.org/10.1007/s11252-011-0204-5.

Ibáñez-Álamo, Juan Diego, and Manuel Soler. 2010. “Does Urbanization Affect Selective Pressures and Life-History Strategies in the Common Blackbird (Turdus Merula L.)?” *Biological Journal of the Linnean Society* 101 (4): 759–66. https://doi.org/10.1111/j.1095-8312.2010.01543.x.

Jarrett, Crinan, Luke L. Powell, Heather McDevitt, Barbara Helm, and Andreanna J. Welch. 2020. “Bitter Fruits of Hard Labour: Diet Metabarcoding and Telemetry Reveal That Urban Songbirds Travel Further for Lower-Quality Food.” *Oecologia* 193 (2): 377–88. https://doi.org/10.1007/s00442-020-04678-w.

Kelleher, Katherine M., and John O’Halloran. 2007. “Influence of Nesting Habitat on Breeding Song Thrushes *Turdus Philomelos*.” *Bird Study* 54 (2): 221–29. https://doi.org/10.1080/00063650709461478.

Kettel, Esther F., Louise K. Gentle, Richard W. Yarnell, and John L. Quinn. 2019. “Breeding Performance of an Apex Predator, the Peregrine Falcon, across Urban and Rural Landscapes.” *Urban Ecosystems* 22 (1): 117–25. https://doi.org/10.1007/s11252-018-0799-x.

Kübler, Sonja, Stefan Kupko, and Ulrich Zeller. 2005. “The Kestrel (Falco Tinnunculus L.) in Berlin: Investigation of Breeding Biology and Feeding Ecology.” *Journal of Ornithology* 146 (3): 271–78. https://doi.org/10.1007/s10336-005-0089-2.

Lin, Wen-Loung, Si-Min Lin, Jhan-Wei Lin, Ying Wang, and Hui-Yun Tseng. 2015. “Breeding Performance of Crested Goshawk *Accipiter Trivirgatus* in Urban and Rural Environments of Taiwan.” *Bird Study* 62 (2): 177–84. https://doi.org/10.1080/00063657.2015.1005570.

Liven-Schulman, Ifat, Yossi Leshem, Dan Alon, and Yoram Yom-Tov. 2004. “Causes BlackwellPublishingLtd. of Population Declines of the Lesser Kestrel Falco Naumanni in Israel.” *Ibis* 146: 145–52.

Marini, Kristen L. D., Ken A. Otter, Stefanie E. LaZerte, and Matthew W. Reudink. 2017. “Urban Environments Are Associated with Earlier Clutches and Faster Nestling Feather Growth Compared to Natural Habitats.” *Urban Ecosystems* 20 (6): 1291–1300. https://doi.org/10.1007/s11252-017-0681-2.

Mazumdar, Abhijit, and Prabhat Kumar. 2014. “Difference in Nesting Ecology of Purple Sunbird Nectarinia Asiatica among Urban and Rural Habitats in New Delhi, India.” *Avocetta* 38: 29–35.

Mennechez, Gwénaëlle, and Philippe Clergeau. 2006. “Effect of Urbanisation on Habitat Generalists: Starlings Not so Flexible?” *Acta Oecologica* 30 (2): 182–91. https://doi.org/10.1016/j.actao.2006.03.002.

Merling De Chapa, Manuela, Alexandre Courtiol, Marc Engler, Lisa Giese, Christian Rutz, Michael Lakermann, Gerard Müskens, et al. 2020. “Phantom of the Forest or Successful Citizen? Analysing How Northern Goshawks ( *Accipiter Gentilis* ) Cope with the Urban Environment.” *Royal Society Open Science* 7 (12): 201356. https://doi.org/10.1098/rsos.201356.

Meyrier, Eva, Lukas Jenni, Yves Bötsch, Stephan Strebel, Bruno Erne, and Zulima Tablado. 2017. “Happy to Breed in the City? Urban Food Resources Limit Reproductive Output in Western Jackdaws.” *Ecology and Evolution* 7 (5): 1363–74. https://doi.org/10.1002/ece3.2733.

Millsap, Brian, Tim Breen, Elizabeth McConnell, Tony Steffer, Laura Phillips, Nancy Douglass, and Sharon Taylor. 2004. “Comparative Fecundity and Survival of Bald Eagles Fledged from Suburban and Rural Natal Areas in Florida.” Edited by Boal. *Journal of Wildlife Management* 68 (4): 1018–31. https://doi.org/10.2193/0022-541X(2004)068[1018:CFASOB]2.0.CO;2.

Minias, Piotr. 2016. “Reproduction and Survival in the City: Which Fitness Components Drive Urban Colonization in a Reed-Nesting Waterbird?” *Current Zoology* 62 (2): 79–87. https://doi.org/10.1093/cz/zow034.

Najmanová, Lenka, and Peter Adamík. 2009. “Effect of Climatic Change on the Duration of the Breeding Season in Three European Thrushes.” *Bird Study* 56 (3): 349–56. https://doi.org/10.1080/00063650902937305.

Pollock, Christopher J., Pablo Capilla-Lasheras, Rona A. R. McGill, Barbara Helm, and Davide M. Dominoni. 2017. “Integrated Behavioural and Stable Isotope Data Reveal Altered Diet Linked to Low Breeding Success in Urban-Dwelling Blue Tits (Cyanistes Caeruleus).” *Scientific Reports* 7 (5014): 1–14. https://doi.org/10.1038/s41598-017-04575-y.

Rollinson, Daniel J., and Darryl N. Jones. 2002. “Variation in Breeding Parameters of the Australian Magpie *Gymnorhina Tibicen* in Suburban and Rural Environments.” *Urban Ecosystems* 6 (4): 257–69. https://doi.org/10.1023/B:UECO.0000004826.52945.ed.

Russ, Anja, Terézia Lučeničová, and Reinhard Klenke. 2017. “Altered Breeding Biology of the European Blackbird under Artificial Light at Night.” *Journal of Avian Biology* 48 (8): 1114–25. https://doi.org/10.1111/jav.01210.

Seress, Gábor, Veronika Bókony, Ivett Pipoly, Tibor Szép, Károly Nagy, and András Liker. 2012. “Urbanization, Nestling Growth and Reproductive Success in a Moderately Declining House Sparrow Population.” *Journal of Avian Biology* 43 (5): 403–14. https://doi.org/10.1111/j.1600-048X.2012.05527.x.

Seress, Gábor, S Hammer, Veronika Bo, Lint Preiszner, Ivett Pipoly, Csenge Sinkovics, Karl L Evans, and S Liker. 2018. “Impact of Urbanization on Abundance and Phenology of Caterpillars and Consequences for Breeding in an Insectivorous Bird.” *Ecological Applications* 28 (5): 1143–56. https://doi.org/10.1002/eap.1730.

Seress, Gábor, Krisztina Sándor, Karl L. Evans, and András Liker. 2020. “Food Availability Limits Avian Reproduction in the City: An Experimental Study on Great Tits *Parus Major*.” Edited by Elizabeth Derryberry. *Journal of Animal Ecology* 89 (7): 1570–80. https://doi.org/10.1111/1365-2656.13211.

Solonen, Tapio. 2001. “Breeding of the Great Tit and Blue Tit in Urban and Rural Habitats in Southern Finland.” *Ornis Fennica* 78: 49–60.

———. 2008. “Larger Broods in the Northern Goshawk Accipiter Gentilis near Urban Areas in Southern Finland.” *Ornis Fennica* 85: 118–25.

Solonen, Tapio, and Martti Hildén. 2014. “Breeding Phenology in Great and Blue Tits (Parus Spp.): Are Urban Populations More Resistant to Climate Change than Rural Ones?” *Ornis Fennica* 91: 209–19.

Solonen, Tapio, Heikki Lokki, and Seppo Sulkava. 2019. “Diet and Brood Size in Rural and Urban Northern Goshawks Accipiter Gentilis in Southern Finland.” *Avian Biology Research* 12 (1): 3–9. https://doi.org/10.1177/1758155919826754.

Solonen, Tapio, and Kimmo Ursin. 2008. “Breeding of Tawny Owls *Strix Aluco* in Rural and Urban Habitats in Southern Finland.” *Bird Study* 55 (2): 216–21. https://doi.org/10.1080/00063650809461525.

Sprau, Philipp, Alexia Mouchet, and Niels J. Dingemanse. 2017. “Multidimensional Environmental Predictors of Variation in Avian Forest and City Life Histories.” *Behavioral Ecology* 28 (1): 59–68. https://doi.org/10.1093/beheco/arw130.

Sumasgutner, Petra, Erwin Nemeth, Graham Tebb, Harald W. Krenn, and Anita Gamauf. 2014. “Hard Times in the City – Attractive Nest Sites but Insufficient Food Supply Lead to Low Reproduction Rates in a Bird of Prey.” *Frontiers in Zoology* 11 (1): 1–14. https://doi.org/10.1186/1742-9994-11-48.

Teglhøj, Peter Györkös. 2017. “A Comparative Study of Insect Abundance and Reproductive Success of Barn Swallows *Hirundo Rustica* in Two Urban Habitats.” *Journal of Avian Biology* 48 (6): 846–53. https://doi.org/10.1111/jav.01086.

Thornton, Michael, Ian Todd, and Staffan Roos. 2017. “Breeding Success and Productivity of Urban and Rural Eurasian Sparrowhawks Accipiter Nisus in Scotland.” *Ecoscience* 24 (3–4): 115–26. https://doi.org/10.1080/11956860.2017.1374322.

Vaugoyeau, Marie, Frank Adriaensen, Alexandr Artemyev, Jerzy Bańbura, Emilio Barba, Clotilde Biard, Jacques Blondel, et al. 2016. “Interspecific Variation in the Relationship between Clutch Size, Laying Date and Intensity of Urbanization in Four Species of Hole‐nesting Birds.” *Ecology and Evolution* 6 (16): 5907–20. https://doi.org/10.1002/ece3.2335.

Wawrzyniak, J., M. Glądalski, A. Kaliński, M. Bańbura, M. Markowski, J. Skwarska, P. Zieliński, and J. Bańbura. 2020. “Differences in the Breeding Performance of Great Tits Parus Major between a Forest and an Urban Area: A Long Term Study on First Clutches.” *The European Zoological Journal* 87 (1): 294–309. https://doi.org/10.1080/24750263.2020.1766125.

Wawrzyniak, Jarosław, Adam Kaliński, Michał Glądalski, Mirosława Bańbura, Marcin Markowski, Joanna Skwarska, Piotr ZielińSki, Iwona Cyżewska, and Jerzy Bańbura. 2015. “Long-Term Variation in Laying Date and Clutch Size of the Great Tit *Parus Major* in Central Poland: A Comparison between Urban Parkland and Deciduous Forest.” *Ardeola* 62 (2): 311–22. https://doi.org/10.13157/arla.62.2.2015.311.

Welch-Acosta, Brandi C., Ben R. Skipper, and Clint W. Boal. 2019. “Comparative Breeding Ecology of Mississippi Kites in Urban and Exurban Areas of West Texas.” *Journal of Field Ornithology* 90 (3): 248–57. https://doi.org/10.1111/jofo.12303.

Whitehouse, Michael J., Nancy M. Harrison, Julia Mackenzie, and Shelley A. Hinsley. 2013. “Preferred Habitat of Breeding Birds May Be Compromised by Climate Change: Unexpected Effects of an Exceptionally Cold, Wet Spring.” *PLOS ONE* 8 (9): e75536. https://doi.org/10.1371/journal.pone.0075536.
