# Supplementary material for: A multidimensional framework to quantify the effects of urbanization on avian breeding fitness
Source: Ecol Evol. 2023 Jul 3;13(7):e10259. doi: 10.1002/ece3.10259 (PMC10316489; doi:10.1002/ece3.10259)
Supplement: Supplementary file 3 — Appendix S3. [file ECE3-13-e10259-s002.docx]

A multidimensional framework to quantify the effects of urbanization on avian breeding fitness

Sihao Chen, Yu Liu, Samantha C. Patrick, Eben Goodale, Rebecca J. Safran, Emilio Pagani-Núñez

Article for Ecology and Evolution

Appendix S3

This document includes detailed information about papers studying urban heat island, noise pollution, artificial light at night and food resources factors. These papers are then categorized based on the methods used in each paper.

Table S1. Studies using urban heat island as an environmental factor

| Grouping Criteria (methods of obtaining data) | Papers |
| --- | --- |
| Meteorological stations or institutions (*N=9)* | (Solonen and Hildén 2014; Jarosław Wawrzyniak et al. 2015; Glądalski et al. 2015; 2016; Kreiderits et al. 2016; Meyrier et al. 2017; Becker and Weisberg 2015; J. Wawrzyniak et al. 2020; Whitehouse et al. 2013) |
| Temperature data logger (*N=1)* | (Sprau, Mouchet, and Dingemanse 2017) |
| Unclear (*N=1)* | (Meyrier et al. 2017) |

Table S2. Studies using noise pollution as an environmental factor

| Grouping Criteria (methods of obtaining data) | Papers |
| --- | --- |
| Playback experiment (*N=5)* | (Schroeder et al. 2012; Potvin and MacDougall-Shackleton 2015; Halfwerk, Both, and Slabbekoorn 2016; Mulholland et al. 2018; Zollinger et al. 2019) |
| Sound pressure level meter (data logger) (*N=7)* | (Halfwerk et al. 2011; Kight, Saha, and Swaddle 2012; Meillere, Brischoux, and Angelier 2015; Sprau, Mouchet, and Dingemanse 2017; Kleist et al. 2018; Injaian, Taff, and Patricelli 2018; Injaian, Poon, and Patricelli 2018) |
| Distance to road (*N=1)* | (Kuitunen et al. 2003) |

Table S3. Studies using artificial light at night as an environmental factor

| Grouping Criteria (methods of obtaining data) | Papers |
| --- | --- |
| Light experiment (*N=1)* | (Dominoni et al. 2020) |
| Light meter (data logger) (*N=1)* | (Sprau, Mouchet, and Dingemanse 2017) |
| Governmental institution (*N=2)* | (Russ, Lučeničová, and Klenke 2017; Jong et al. 2018) |
| Online data source (*N=1)* | (Jong et al. 2018) |

Table S4. Studies using food abundance as a factor

| Grouping Criteria (methods of obtaining data) | Papers |
| --- | --- |
| Food-supplemented experiment (*N=11)* | (Harrison et al. 2010; Meyrier et al. 2017; Salleh Hudin et al. 2017; Demeyrier et al. 2017; Seress et al. 2020; Peach, Sheehan, and Kirby 2014; Styrsky, Dobbs, and Thompson 2000; Reynolds, Schoech, and Bowman 2003; Pearse, Cavitt, and Cully 2004; De Neve et al. 2004; Clinchy et al. 2004) |
| Frassfall collecting method (*N=7)* | (Solonen 2001; Glądalski et al. 2015; 2017; Jarosław Wawrzyniak et al. 2015; J. Wawrzyniak et al. 2020; Seress et al. 2018; Hajdasz et al. 2019) |
| Pellet collecting method (*N=3)* | (Kübler, Kupko, and Zeller 2005; Sumasgutner et al. 2014; Solonen, Lokki, and Sulkava 2019) |
| Other unconventional methods (*N=4)* | (Stock and Haag-Wackernagel 2016; Pollock et al. 2017; Kettel et al. 2019; Tortosa, Pérez, and Hillström 2003) |

LITERATURE CITED

Becker, Miles E., and Peter J. Weisberg. 2015. “Synergistic Effects of Spring Temperatures and Land Cover on Nest Survival of Urban Birds.” *The Condor* 117 (1): 18–30. https://doi.org/10.1650/CONDOR-14-1.1.

Clinchy, Michael, Liana Zanette, Rudy Boonstra, John C. Wingfield, and James N. M. Smith. 2004. “Balancing Food and Predator Pressure Induces Chronic Stress in Songbirds.” *Proceedings of the Royal Society B: Biological Sciences* 271 (1556): 2473–79. https://doi.org/10.1098/rspb.2004.2913.

De Neve, Liesbeth, Juan J. Soler, Manuel Soler, Tomás Pérez-Contreras, Manuel Martín-Vivaldi, and Juan G. Martínez. 2004. “Effects of a Food Supplementation Experiment on Reproductive Investment and a Post-Mating Sexually Selected Trait in Magpies *Pica Pica*.” *Journal of Avian Biology* 35 (3): 246–51. https://doi.org/10.1111/j.0908-8857.2004.03162.x.

Demeyrier, Virginie, Anne Charmantier, Marcel M. Lambrechts, and Arnaud Grégoire. 2017. “Disentangling Drivers of Reproductive Performance in Urban Great Tits: A Food Supplementation Experiment.” *Journal of Experimental Biology* 220 (22): 4195–4203. https://doi.org/10.1242/jeb.161067.

Dominoni, Davide M., Johan Kjellberg Jensen, Maaike Jong, Marcel E. Visser, and Kamiel Spoelstra. 2020. “Artificial Light at Night, in Interaction with Spring Temperature, Modulates Timing of Reproduction in a Passerine Bird.” *Ecological Applications* 30 (3): e02062. https://doi.org/10.1002/eap.2062.

Glądalski, Michał, Mirosława Bańbura, Adam Kaliński, Marcin Markowski, Joanna Skwarska, Jarosław Wawrzyniak, Piotr Zieliński, and Jerzy Bańbura. 2016. “Effects of Extreme Thermal Conditions on Plasticity in Breeding Phenology and Double-Broodedness of Great Tits and Blue Tits in Central Poland in 2013 and 2014.” *International Journal of Biometeorology* 60 (11): 1795–1800. https://doi.org/10.1007/s00484-016-1152-9.

Glądalski, Michał, Miroslawa Banbura, Adam Kalinski, Marcin Markowski, Joanna Skwarska, Jaroslaw Wawrzyniak, Piotr Zielinski, Iwona Cyzewska, and Jerzy Banbura. 2015. “Inter-Annual and Inter-Habitat Variation in Breeding Performance of Blue Tits (Cyanistes Caeruleus) in Central Poland.” *Ornis Fennica* 92: 34–42.

Glądalski, Michał, Mirosława Bańbura, Adam Kaliński, Marcin Markowski, Joanna Skwarska, Jarosław Wawrzyniak, Piotr Zieliński, Iwona Cyżewska, and Jerzy Bańbura. 2017. “Differences in the Breeding Success of Blue Tits *Cyanistes Caeruleus* between a Forest and an Urban Area: A Long-Term Study.” *Acta Ornithologica* 52 (1): 59–68. https://doi.org/10.3161/00016454AO2017.52.1.006.

Hajdasz, Adrianne C., Ken A. Otter, Lyn K. Baldwin, and Matthew W. Reudink. 2019. “Caterpillar Phenology Predicts Differences in Timing of Mountain Chickadee Breeding in Urban and Rural Habitats.” *Urban Ecosystems* 22 (6): 1113–22. https://doi.org/10.1007/s11252-019-00884-4.

Halfwerk, Wouter, Christiaan Both, and Hans Slabbekoorn. 2016. “Noise Affects Nest-Box Choice of 2 Competing Songbird Species, but Not Their Reproduction.” *Behavioral Ecology* 27 (6): 1592–1600. https://doi.org/10.1093/beheco/arw095.

Halfwerk, Wouter, Leonard J. M. Holleman, C(Kate) M. Lessells, and Hans Slabbekoorn. 2011. “Negative Impact of Traffic Noise on Avian Reproductive Success.” *Journal of Applied Ecology* 48 (1): 210–19. https://doi.org/10.1111/j.1365-2664.2010.01914.x.

Harrison, Timothy J. E., Jennifer A. Smith, Graham R. Martin, Dan E. Chamberlain, Stuart Bearhop, Gillian N. Robb, and S. James Reynolds. 2010. “Does Food Supplementation Really Enhance Productivity of Breeding Birds?” *Oecologia* 164 (2): 311–20. https://doi.org/10.1007/s00442-010-1645-x.

Injaian, Allison S, Lauren Y Poon, and Gail L Patricelli. 2018. “Effects of Experimental Anthropogenic Noise on Avian Settlement Patterns and Reproductive Success.” *Behavioral Ecology* 29 (5): 1181–89. https://doi.org/10.1093/beheco/ary097.

Injaian, Allison S., Conor C. Taff, and Gail L. Patricelli. 2018. “Experimental Anthropogenic Noise Impacts Avian Parental Behaviour, Nestling Growth and Nestling Oxidative Stress.” *Animal Behaviour* 136 (February): 31–39. https://doi.org/10.1016/j.anbehav.2017.12.003.

Jong, Maaike de, Laura van den Eertwegh, Ronald E. Beskers, Peter P. de Vries, Kamiel Spoelstra, and Marcel E. Visser. 2018. “Timing of Avian Breeding in an Urbanised World.” *Ardea* 106 (1): 31–38. https://doi.org/10.5253/arde.v106i1.a4.

Kettel, Esther F., Louise K. Gentle, Richard W. Yarnell, and John L. Quinn. 2019. “Breeding Performance of an Apex Predator, the Peregrine Falcon, across Urban and Rural Landscapes.” *Urban Ecosystems* 22 (1): 117–25. https://doi.org/10.1007/s11252-018-0799-x.

Kight, Caitlin R., Margaret S. Saha, and John P. Swaddle. 2012. “Anthropogenic Noise Is Associated with Reductions in the Productivity of Breeding Eastern Bluebirds ( *Sialia Sialis* ).” *Ecological Applications* 22 (7): 1989–96. https://doi.org/10.1890/12-0133.1.

Kleist, Nathan J., Robert P. Guralnick, Alexander Cruz, Christopher A. Lowry, and Clinton D. Francis. 2018. “Chronic Anthropogenic Noise Disrupts Glucocorticoid Signaling and Has Multiple Effects on Fitness in an Avian Community.” *Proceedings of the National Academy of Sciences* 115 (4): 648–57. https://doi.org/10.1073/pnas.1709200115.

Kreiderits, A., A. Gamauf, H. W. Krenn, and P. Sumasgutner. 2016. “Investigating the Influence of Local Weather Conditions and Alternative Prey Composition on the Breeding Performance of Urban Eurasian Kestrels Falco Tinnunculus.” *Bird Study* 63 (3): 369–79. https://doi.org/10.1080/00063657.2016.1213791.

Kübler, Sonja, Stefan Kupko, and Ulrich Zeller. 2005. “The Kestrel (Falco Tinnunculus L.) in Berlin: Investigation of Breeding Biology and Feeding Ecology.” *Journal of Ornithology* 146 (3): 271–78. https://doi.org/10.1007/s10336-005-0089-2.

Kuitunen, Markku T., Johanna Viljanen, Esko Rossi, and Arto Stenroos. 2003. “Impact of Busy Roads on Breeding Success in Pied Flycatchers Ficedula Hypoleuca.” *Environmental Management* 31 (1): 79–85. https://doi.org/10.1007/s00267-002-2694-7.

Meillere, A., F. Brischoux, and F. Angelier. 2015. “Impact of Chronic Noise Exposure on Antipredator Behavior: An Experiment in Breeding House Sparrows.” *Behavioral Ecology* 26 (2): 569–77. https://doi.org/10.1093/beheco/aru232.

Meyrier, Eva, Lukas Jenni, Yves Bötsch, Stephan Strebel, Bruno Erne, and Zulima Tablado. 2017. “Happy to Breed in the City? Urban Food Resources Limit Reproductive Output in Western Jackdaws.” *Ecology and Evolution* 7 (5): 1363–74. https://doi.org/10.1002/ece3.2733.

Mulholland, Tracy I, Danielle M Ferraro, Kelley C Boland, Kathleen N Ivey, My-Lan Le, Carl A LaRiccia, John M Vigianelli, and Clinton D Francis. 2018. “Effects of Experimental Anthropogenic Noise Exposure on the Reproductive Success of Secondary Cavity Nesting Birds.” *Integrative and Comparative Biology* 58 (5): 967–76. https://doi.org/10.1093/icb/icy079.

Peach, Will J., Danaë K. Sheehan, and Will B. Kirby. 2014. “Supplementary Feeding of Mealworms Enhances Reproductive Success in Garden Nesting House Sparrows *Passer Domesticus*.” *Bird Study* 61 (3): 378–85. https://doi.org/10.1080/00063657.2014.918577.

Pearse, Aaron T., John F. Cavitt, and Jack F. Cully. 2004. “Effects of Food Supplementation on Female Nest Attentiveness and Incubation Mate Feeding in Two Sympatric Wren Species.” *The Wilson Bulletin* 116 (1): 23–30. https://doi.org/10.1676/0043-5643(2004)116[0023:EOFSOF]2.0.CO;2.

Pollock, Christopher J., Pablo Capilla-Lasheras, Rona A. R. McGill, Barbara Helm, and Davide M. Dominoni. 2017. “Integrated Behavioural and Stable Isotope Data Reveal Altered Diet Linked to Low Breeding Success in Urban-Dwelling Blue Tits (Cyanistes Caeruleus).” *Scientific Reports* 7 (5014): 1–14. https://doi.org/10.1038/s41598-017-04575-y.

Potvin, Dominique A., and Scott A. MacDougall-Shackleton. 2015. “Traffic Noise Affects Embryo Mortality and Nestling Growth Rates in Captive Zebra Finches.” *Journal of Experimental Zoology Part A: Ecological Genetics and Physiology* 323 (10): 722–30. https://doi.org/10.1002/jez.1965.

Reynolds, S. James, Stephan J. Schoech, and Reed Bowman. 2003. “Diet Quality during Pre‐laying and Nestling Periods Influences Growth and Survival of Florida Scrub‐jay ( *Aphelocoma Coerulescens* ) Chicks.” *Journal of Zoology* 261 (3): 217–26. https://doi.org/10.1017/S0952836903004023.

Russ, Anja, Terézia Lučeničová, and Reinhard Klenke. 2017. “Altered Breeding Biology of the European Blackbird under Artificial Light at Night.” *Journal of Avian Biology* 48 (8): 1114–25. https://doi.org/10.1111/jav.01210.

Salleh Hudin, Noraine, Liesbeth De Neve, Diederik Strubbe, Graham D. Fairhurst, Carl Vangestel, Will J. Peach, and Luc Lens. 2017. “Supplementary Feeding Increases Nestling Feather Corticosterone Early in the Breeding Season in House Sparrows.” *Ecology and Evolution* 7 (16): 6163–71. https://doi.org/10.1002/ece3.3114.

Schroeder, Julia, Shinichi Nakagawa, Ian R. Cleasby, and Terry Burke. 2012. “Passerine Birds Breeding under Chronic Noise Experience Reduced Fitness.” *PLOS ONE* 7 (7): e39200. https://doi.org/10.1371/journal.pone.0039200.

Seress, Gábor, S Hammer, Veronika Bo, Lint Preiszner, Ivett Pipoly, Csenge Sinkovics, Karl L Evans, and S Liker. 2018. “Impact of Urbanization on Abundance and Phenology of Caterpillars and Consequences for Breeding in an Insectivorous Bird.” *Ecological Applications* 28 (5): 1143–56. https://doi.org/10.1002/eap.1730.

Seress, Gábor, Krisztina Sándor, Karl L. Evans, and András Liker. 2020. “Food Availability Limits Avian Reproduction in the City: An Experimental Study on Great Tits *Parus Major*.” Edited by Elizabeth Derryberry. *Journal of Animal Ecology* 89 (7): 1570–80. https://doi.org/10.1111/1365-2656.13211.

Solonen, Tapio. 2001. “Breeding of the Great Tit and Blue Tit in Urban and Rural Habitats in Southern Finland.” *Ornis Fennica* 78: 49–60.

Solonen, Tapio, and Martti Hildén. 2014. “Breeding Phenology in Great and Blue Tits (Parus Spp.): Are Urban Populations More Resistant to Climate Change than Rural Ones?” *Ornis Fennica* 91: 209–19.

Solonen, Tapio, Heikki Lokki, and Seppo Sulkava. 2019. “Diet and Brood Size in Rural and Urban Northern Goshawks Accipiter Gentilis in Southern Finland.” *Avian Biology Research* 12 (1): 3–9. https://doi.org/10.1177/1758155919826754.

Sprau, Philipp, Alexia Mouchet, and Niels J. Dingemanse. 2017. “Multidimensional Environmental Predictors of Variation in Avian Forest and City Life Histories.” *Behavioral Ecology* 28 (1): 59–68. https://doi.org/10.1093/beheco/arw130.

Stock, Birte, and Daniel Haag-Wackernagel. 2016. “Food Shortage Affects Reproduction of Feral Pigeons Columba Livia at Rearing of Nestlings.” *Ibis* 158 (4): 776–83. https://doi.org/10.1111/ibi.12385.

Styrsky, John D., Robert C. Dobbs, and Charles F. Thompson. 2000. “Food-Supplementation Does Not Override the Effect of Egg Mass on Fitness-Related Traits of Nestling House Wrens.” *Journal of Animal Ecology* 69 (4): 690–702. https://doi.org/10.1046/j.1365-2656.2000.00427.x.

Sumasgutner, Petra, Erwin Nemeth, Graham Tebb, Harald W. Krenn, and Anita Gamauf. 2014. “Hard Times in the City – Attractive Nest Sites but Insufficient Food Supply Lead to Low Reproduction Rates in a Bird of Prey.” *Frontiers in Zoology* 11 (1): 1–14. https://doi.org/10.1186/1742-9994-11-48.

Tortosa, Francisco S., Lorenzo Pérez, and Lars Hillström. 2003. “Effect of Food Abundance on Laying Date and Clutch Size in the White Stork *Ciconia Ciconia*.” *Bird Study* 50 (2): 112–15. https://doi.org/10.1080/00063650309461302.

Wawrzyniak, J., M. Glądalski, A. Kaliński, M. Bańbura, M. Markowski, J. Skwarska, P. Zieliński, and J. Bańbura. 2020. “Differences in the Breeding Performance of Great Tits Parus Major between a Forest and an Urban Area: A Long Term Study on First Clutches.” *The European Zoological Journal* 87 (1): 294–309. https://doi.org/10.1080/24750263.2020.1766125.

Wawrzyniak, Jarosław, Adam Kaliński, Michał Glądalski, Mirosława Bańbura, Marcin Markowski, Joanna Skwarska, Piotr ZielińSki, Iwona Cyżewska, and Jerzy Bańbura. 2015. “Long-Term Variation in Laying Date and Clutch Size of the Great Tit *Parus Major* in Central Poland: A Comparison between Urban Parkland and Deciduous Forest.” *Ardeola* 62 (2): 311–22. https://doi.org/10.13157/arla.62.2.2015.311.

Whitehouse, Michael J., Nancy M. Harrison, Julia Mackenzie, and Shelley A. Hinsley. 2013. “Preferred Habitat of Breeding Birds May Be Compromised by Climate Change: Unexpected Effects of an Exceptionally Cold, Wet Spring.” *PLOS ONE* 8 (9): e75536. https://doi.org/10.1371/journal.pone.0075536.

Zollinger, Sue Anne, Adriana Dorado-Correa, Wolfgang Goymann, Wolfgang Forstmeier, Ulrich Knief, Ana María Bastidas­Urrutia, and Henrik Brumm. 2019. “Traffic Noise Exposure Depresses Plasma Corticosterone and Delays Offspring Growth in Breeding Zebra Finches.” *Conservation Physiology* 7 (January): 1–15. https://doi.org/10.1093/conphys/coz056.
