# Supplementary material for: A multidimensional framework to quantify the effects of urbanization on avian breeding fitness
Source: Ecol Evol. 2023 Jul 3;13(7):e10259. doi: 10.1002/ece3.10259 (PMC10316489; doi:10.1002/ece3.10259)
Supplement: Supplementary file 4 — Appendix S4. [file ECE3-13-e10259-s003.docx]

A multidimensional framework to quantify the effects of urbanization on avian breeding fitness

Sihao Chen, Yu Liu, Samantha C. Patrick, Eben Goodale, Rebecca J. Safran, Emilio Pagani-Núñez

Article for Ecology and Evolution

Appendix S4

This appendix includes attribution for figures.

Table S1. Attributions for Icons used in Figure 4 under CC BY 3.0. All icons are from thenounproject.com.

| 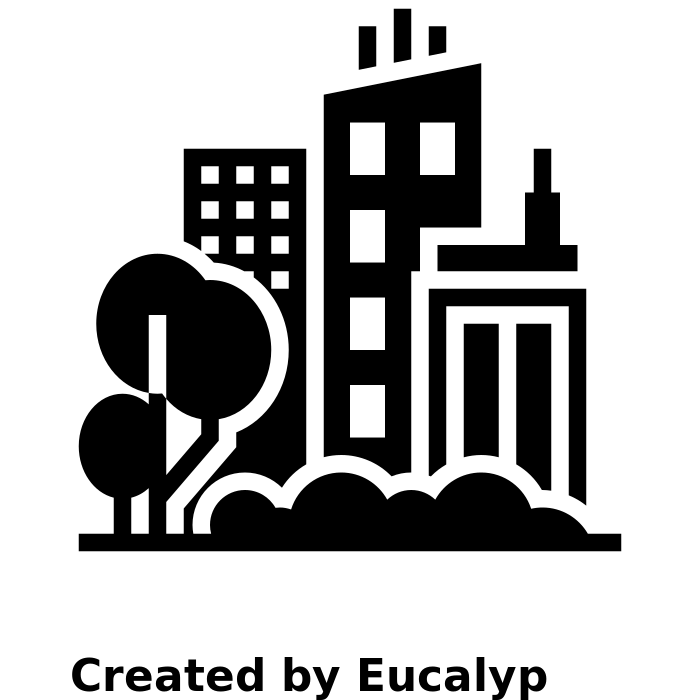 | Urban Design #3156713 created by Eucalyp and changes were made  https://thenounproject.com/icon/urban-design-3156713/ |
| --- | --- |
| 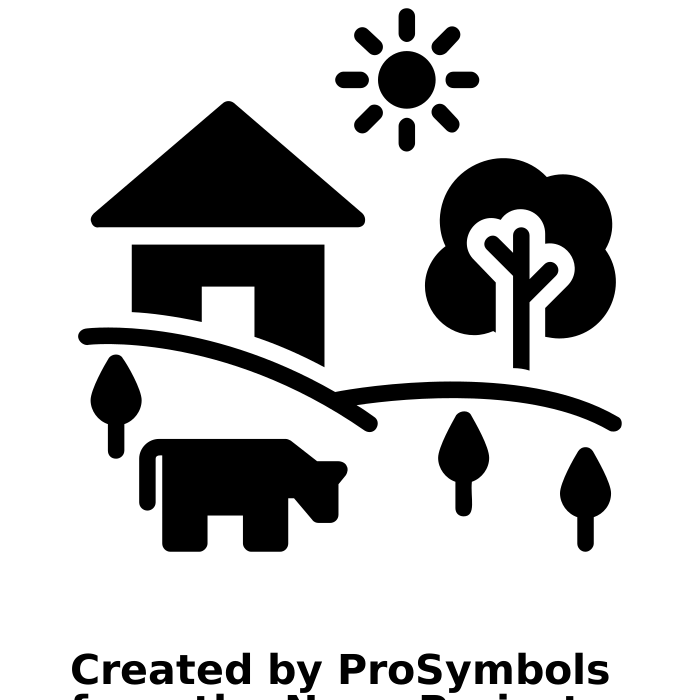 | Farm House #2122755 created by ProSymbols and changes were made  https://thenounproject.com/icon/farm-house-2122755/ |
| 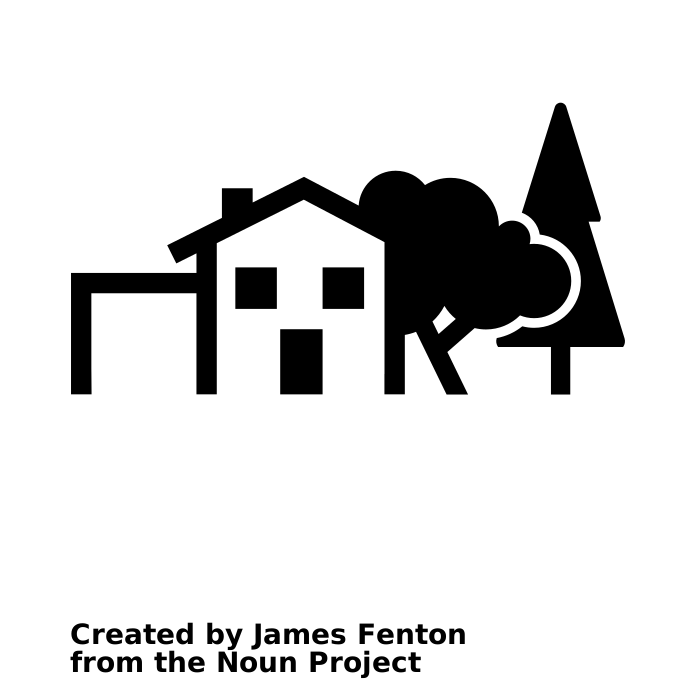 | House #21863 created by James Fenton  https://thenounproject.com/icon/house-21863/ |
| 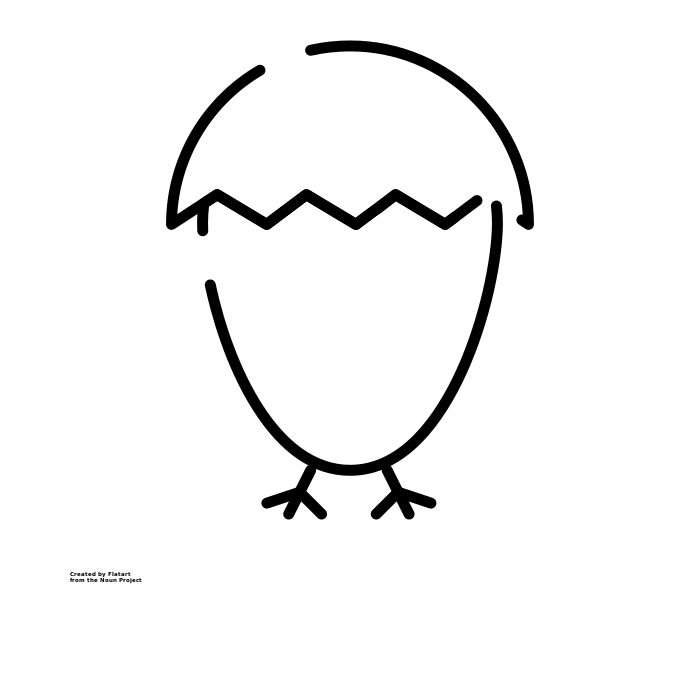 | Bird #2395205 Flatart  https://thenounproject.com/99277952/settings/icon-history/ |
| 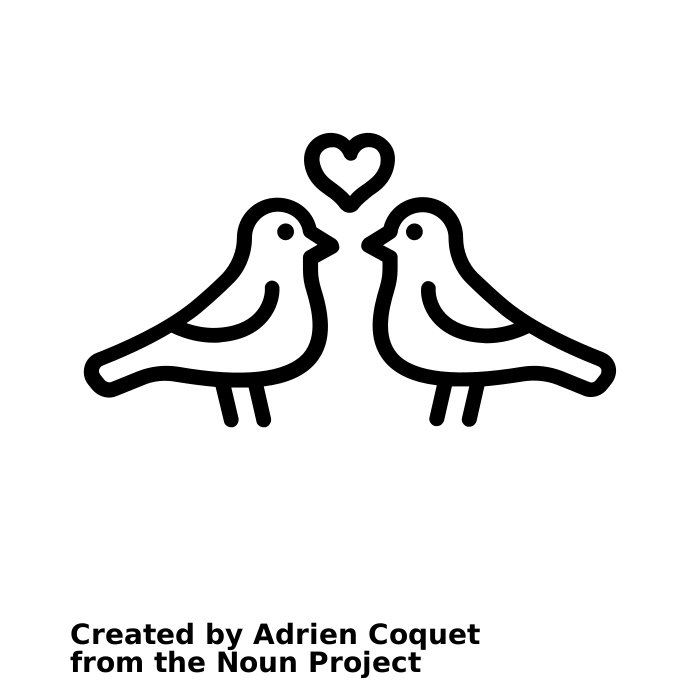 | Birds #4374264 Adrien Coquet  https://thenounproject.com/icon/birds-4374264/ |
| 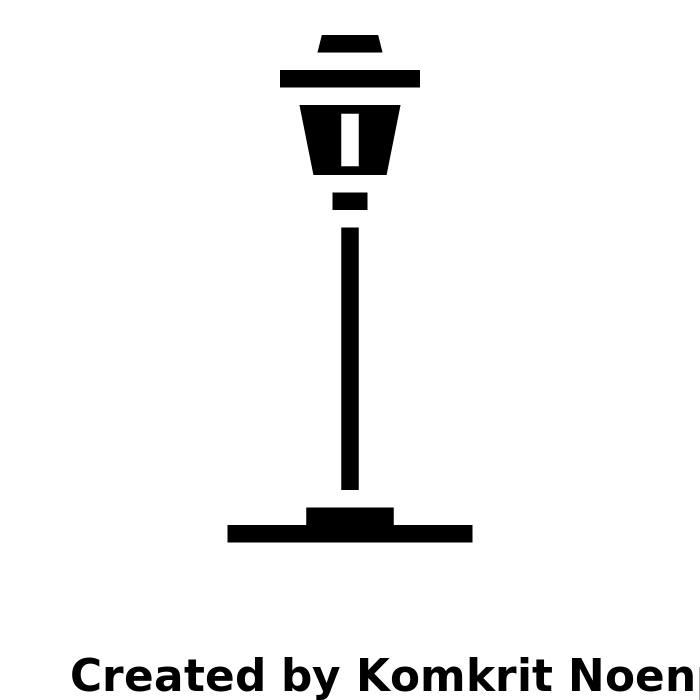 | Night Light #3471773 Komkrit Noenpoempisut  https://thenounproject.com/icon/night-light-3471773/ |
| 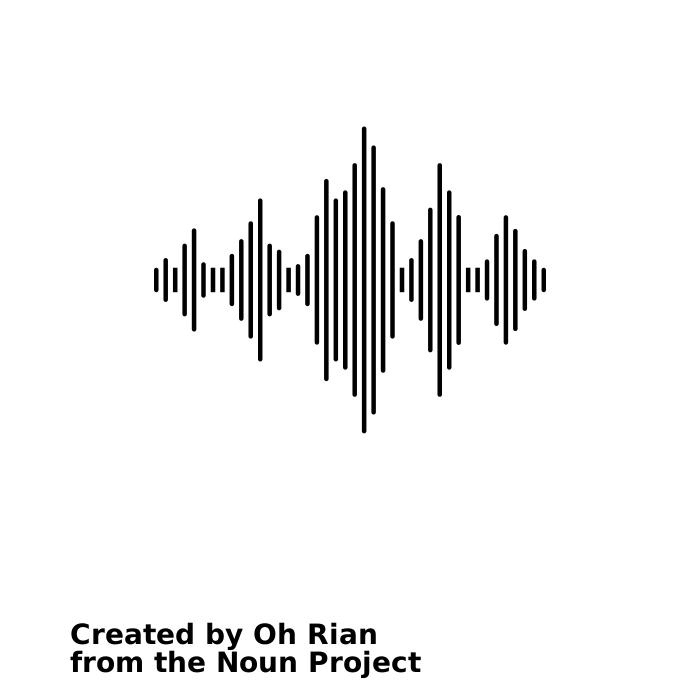 | Noise #4209887 Oh Rian  https://thenounproject.com/icon/noise-4209887/ |
| 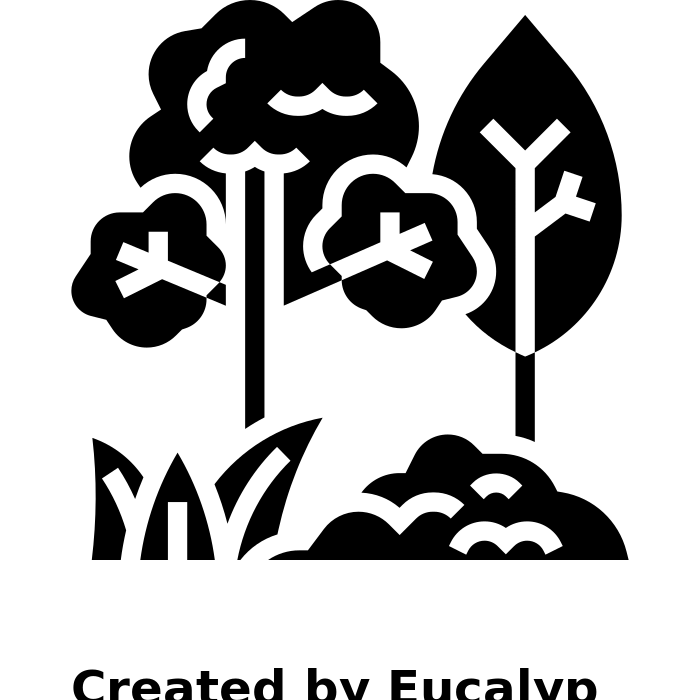 | Forest #4168292 Eucalyp  https://thenounproject.com/icon/forest-4168292/ |
| 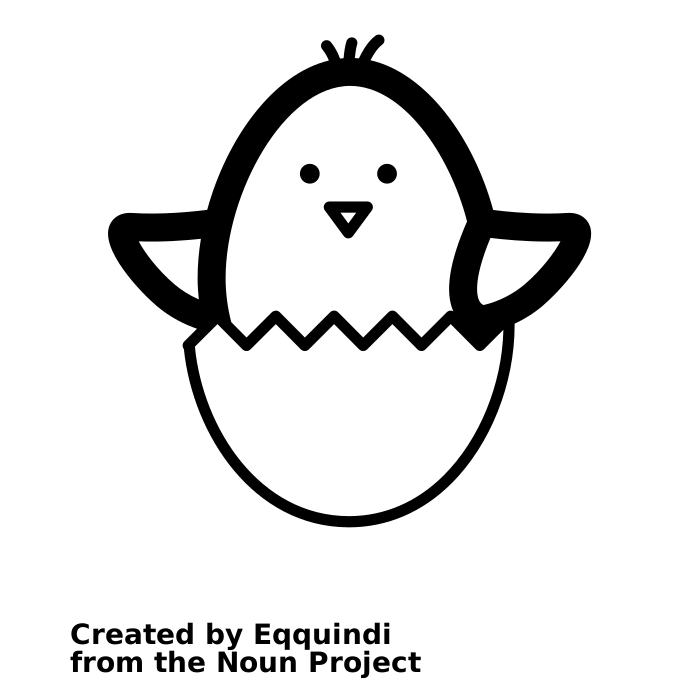 | Chick #1654575 Eqquindi  https://thenounproject.com/icon/chick-1654575/ |
| 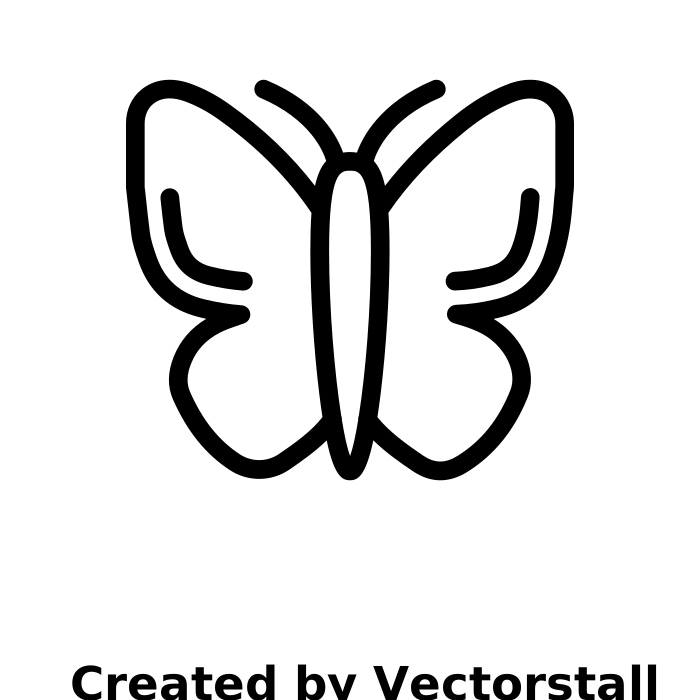 | Insect #3714477 Vectorstall  https://thenounproject.com/icon/insect-3714477/ |
